# The impact of non-environmental factors on the chemical variation of Radix S*crophulariae*

**DOI:** 10.1016/j.heliyon.2024.e24468

**Published:** 2024-01-12

**Authors:** Hui Yao, Jian Sun, Mengying Chen, Yu Dong, Pan Wang, Jianzhong Xu, Qingsong Shao, Zhian Wang

**Affiliations:** a School of Pharmaceutical Sciences, Zhejiang Chinese Medical University, Hangzhou, 310053, China; b Zhejiang Research Institute of Traditional Chinese Medicine Co., Ltd., Hangzhou, 310023, China; c Zhejiang Provincial Key Laboratory of Resources Protection and Innovation of Traditional Chinese Medicine, Zhejiang Agriculture & Forest University, Hangzhou, 311300, China; d Zhejiang Academy of Traditional Chinese Medicine, Hangzhou, 310007, China; e Institute of Traditional Chinese Medicine Industry Innovation of Pan'an, Pan'an, 322300, China

**Keywords:** *Scrophularia ningpoensis* hemsl., Non-environmental factors, SRAP, SSR, HPLC

## Abstract

Radix *Scrophulariae* is a commonly used Chinese herb derived from the dried root of *Scrophularia ningpoesis* Hemsl. (*S. ningpoensis*). It is difficult to accurately estimate the dosage of Chinese medicinal materials used in the prescription because of the chemical variation caused by various factors. To analyze the non-environmental factors affecting the chemical variation of Radix *Scrophulariae*, we planted nine different cultivated varieties of *S. ningpoensis* in the same plantation. Based on sequence-related amplified polymorphism (SRAP), simple sequence repeats (SSR) markers and high-performance liquid chromatography (HPLC) analysis, we found that the materials from the cultivated varieties could be divided into two groups, the Zhejiang group, and the southwest China group. The genetic distance based on molecular data between the two groups was above 0.3882, and the Euclidean distance based on chemical data between the two groups was above 5.312. The correlation analysis between the genetic distance matrix based on SRAP and the Euclidean distance matrix based on 18 HPLC peaks of the whole underground part revealed that the genetic differentiation and chemical variation were positively related, r = 0.7196 (p < 0.05). The genetic background, different part of the roots and the different development of the roots are the three non-environmental factors causing the chemical variation. The coefficient of variation (*C.V*) of chemical composition of Radix *Scrophulariae* with different genetic background reached to 93.62 %, the *C.V* of the chemical composition of Radix *Scrophulariae* derived from the same variety reached to 64.21 %, the *C.V* of the chemical composition of Radix *Scrophulariae* derived from the middle part of the roots of *S. ningpoensis* from the same variety reached to 45.55 %. The *C.V* of chemical composition of Radix *Scrophulairae* produced in the same plantation could be controlled to 38.43 % by using the same variety of roots with the approximate mass derived from the middle part of the roots under 20 g. Our findings provided insights to decrease the chemical variation of Chinese medicinal materials by controlling non-environmental factors.

## 1 Introduction

Radix *Scrophulariae* derived from the perennial herb *Scrophularia ningpoensis* Hemsl. Has a long history of clinical application in China which is included in the *Chinese Pharmacopoeia* (2020 edition) and recorded in *Shennong's Herbal Classic of Materia Medica*. Wild *S*. *ningpoensis* is widely distributed in Hebei, Henan, Shaanxi, Hubei, Anhui, Zhejiang, and other provinces in China. Radix *Scrophulariae* on the market mainly comes from the two main cultivation areas in China which are the middle of Zhejiang province and southwest China including the border joint areas among administrative regions of Hubei, Hunan, Guizhou, and Chongqing.

Radix *Scrophulariae* contains many active components including iridoids, phenylpropanoids, organic acids, volatile oils, terpenoids, steroids, flavonoids, nucleosides et al. Among them, iridoids are considered as the main active components [1]. Chen et al. [2] found that harbagide and harbagoside could alleviate myocardial damage in rats by inhibiting the increase of Ca^2+^ concentration induced by KCl. Liu et al. [3] found that peach leaf corallin could effectively improve the survival rate of human normal liver HL-7702 cells and thus played an important role in liver protection. Tian et al. [4] found that aucubin, harpagide and harpagoside all had hypoglycemic activity on human hepatocellular carcinoma HepG2 cells. Besides iridoid, phenylpropanoids are another major component of Radix *Scrophulariae* including angoroside C, acteoside and cinnamic acid, which have anti-platelet agglutination [5], uric acid lowering [6], antioxidant activity [7] and anti-inflammatory [8] effects, respectively.

Due to low cost and reliable data, sequence-related amplified polymorphism molecular markers (SRAP) have been widely used in the genetic diversity of medicinal plants, such as *Polygonatum* spp [9]. and *Citrus* [10]. Chen et al. [11] found that SRAP could well analyze the genetic diversity of three cultivated populations of *S. ningpoensis*. Simple repeat sequence (SSR) molecular markers have the advantages of rich polymorphism, good repeatability, co-dominant inheritance, and easy manipulation, and are still widely used in germplasm identification, genetic diversity analysis and genetic relationship analysis [[12], [13], [14], [15], [16]].

The stability of traditional Chinese medicine efficacy is easily affected by many factors such as genetic differentiation, production environment and processing technology. Medina-Holguin et al. [17] found that the chemical variation in the roots of *Anemopsis californica* in different populations was mainly caused by its genetic differentiation. Hu et al. [18] found that the differences in chemical composition were closely related to the rich genetic diversity of different populations. Tahir et al. [19] found that the clustering results of genetic diversity and chemical composition differences of maize in three producing areas of Iraq were consistent. Kadu et al. [20] found that the genetic distance matrix of *Prunus africana* was positively correlated with the Euclidean distance matrix composed of chemical components. These studies demonstrated the feasibility of molecular markers in combination with high-performance liquid chromatography (HPLC) for analyzing the correlation between genetic variation and quality of medicinal plants. The environmental factors such as temperature, humidity and precipitation in different period of a year can also affect the quality of the *S. ningpoensis*. Yang et al. [21] revealed that harpagoside variation was strongly positively correlated with changes of temperature. Tong et al. [22] discovered that the content of different metabolites of Radix *Scrophualrariae* were negatively corellated with humidity and precipitation in October, and positively correlated with evaporation and surface temperature. But the non-environmental factors including genetic, different parts of the underground part and mass of the root are not systematically discussed.

Numerous studies have been conducted focusing on genetic, chemical and pharmacology of *S. ningpoensis*, but few have been concentrated on the non-environmental factors including both the differentiation in genetic and physical parameters of the medicine materials that cause variation in chemical composition.

In this study, SRAP and SSR markers were used to analyze the genetic diversity of *S. ningpoensis* from different varieties. For analysis of the chemical composition among different varieties, roots of different mass of the same variety, and different parts of the whole underground part of *S. ningpoensis*, we constructed HPLC fingerprints and simultaneous quantified the content of six components (aucubin, harpagide, acteoside, angoroside C, harpagoside and cinnamic acid). The effects of non-environmental factors on the quality variation of Radix *Scrophulariae* were systematically discussed, which provided a useful strategy for further quality control and product development of Radix *Scrophulariae*. The workflow of this study is shown in Fig. S1.

## 2 Materials and methods

### 2.1 Plant materials and genomic DNA extraction

**Fig. 1:**
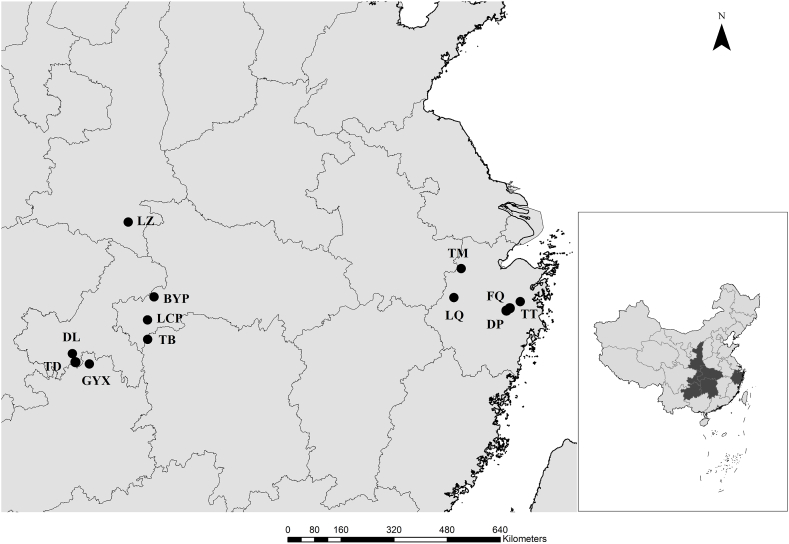
Distribution of studied populations of *S. ningpoensis.* Different dots represent the original locations of the studied *S. ningpoensis* (Explanation see Table 1).

In November of 2021, a total of nine cultivated *S. ningpoensis* varieties were collected from southwest China (the border joint areas of Hubei, Hunan, Guizhou, Chongqing, and Shaanxi) and Zhejiang Province (Fig. 1). The samples’ basic information is shown in Table 1. More than ten well-developed single plants were collected from each of the nine cultivated varieties of *S. ningpoensis.* And in December, ten basal buds of each varieties in white color with moderate size around 0.6–1.0 cm in diameter and 3–5 cm in length were planted with planting density of 40 cm × 50 cm and planting depth of 5–15 cm in Hangzhou city, Zhejiang Province (Latitude: 119° 58′; Longitude: 30° 22′), the methodology of panting, filed management and fertilization refers to the research of Li [23]. And the harvesting time was November 8th of 2022. In addition, three wild accessions were collected for genetic analysis from Hangzhou, Taizhou, and Chun ‘an of Zhejiang Province in November of 2022.

**Table 1:** The basic origin information of studied *S. ningpoensis*.

ID	Classification	Origin	Latitude (N)	Longitude €	Altitude(m)
TM	Wild accession from Zhejiang	Tianmu Mountain, L'n ‘an District, Hangzhou City, Zhejiang Province	119°26′	30°21′	1086
TT	Wild accession l from Zhejiang	Tiantai Mountain, Tiantai County, Taizhou City, Zhejiang Province	121°02′	29°09′	122
LQ	Wild accession from Zhejiang	Linqi Town, Ch'n ‘an County, Hangzhou City, Zhejiang Province	119°07′	29°51′	253
FQ	Cultivated variety from Zhejiang	Fangqian Town, PananCounty, Jinhua city, Zhejiang Province	120°41′	29°02′	178
DP	Cultivated variety from Zhejiang	Dapan town, Panan County, Jinhua city, Zhejiang Province	120’34′	29°00′	638
LZ	Cultivated variety from Southwest	Laozhuang Town, Zhenping County, Ankang City, Shaanxi Province	112°17′	33°08′	1186
BYP	Cultivated variety from Southwest	Baiyangping Town, Jianshi County, Enshi Tujia Miao Autonomous Prefecture, Hubei Province	109°30′	30°16′	1124
TB	Cultivated variety from Southwest	Shuitianba Town, Longshan County, Xiangxi Tujia and Miao Autonomous Prefecture, Hunan Province	109°43′	29°27′	638
LCP	Cultivated variety from Southwest	Lvcongpo Town, Badong County, Enshi Tujia Miao Autonomous Prefecture, Hubei Province	109°30′	30°16′	1353
DL	Cultivated variety from Southwest	Delong Town, Nanchuan District, Chongqing City	107°15′	28°55′	1213
TD	Cultivated variety from Southwest	Toudu Town, Nanchuan District, Chongqing City	107°11′	28°55′	1259
GYX	Cultivated variety from Southwest	Yangxi Town, Daozhen County, Zunyi City, Guizhou Province	107°35′	29°05′	964

The genomic DNA at the concentration of 100–150 ng was isolated from fresh dried leaves which were ground by TissueLyser Type II tissue grinder (Qiagen, Germany) by Hexadecyl Trimethyl Ammonium Bromide (CTAB) method [24]. DNA quality and concentration were detected by a M200 PRO Multimode Microplate Reader (Tecan, USA). Each DNA sample was stored at −4 °C.

### 2.2 PCR amplification

The SRAP primers used in this study were developed by Li and Quiros [25]. Transcriptome sequencing was conducted on the root tissues of *Scrophularia ningpoensis* Hemsl. RNA extraction was carried out using the Trizol reagent (Invitrogen, CA, USA). Hangzhou Lianchuan Biotechnology Co., Ltd utilized the Illumina Hiseq 4000 platform to generate RNA-seq data containing raw reads. Reads from the three separate libraries were pooled for assembly, followed by normalization to obtain the unigenes. SSR screening was conducted using MicroSatellite (MISA) within the identified unigenes of the transcriptome. The search encompassed three, four, five, and six nucleotide repetitive unigenes. The software Primer 5 was used to design SSR-specific primers [26]. Primer selection criteria included a repeat unit of three bases with only one repeat type. The fragment length ranged from greater than 100 bp and less than 400 bp. 20–23 bp with a TM value of 55°. The number of base repeats was restricted to more than or equal to five. To obtained highly reproducible polymorphism markers, 88 SRAP primer combinations and 200 SSRs were screened across 9 cultivated varieties and 3 wild accessions. Polymerase Chain Reaction (PCR) of each sample was performed in 10 μL volume containing 6 μL 2 × HieffTM PCR Master Mix (Shanghai Yisheng Biotechnology Co., LTD.), 1 μL forward primer, 1 μL reverse primer, 1 μL DNA and 1 μL ddH_2_O. PCR amplification modified from Feng et al. [27] was performed on Veriti 96 Well Thermal Cycler PCR instrument (Thermo Fisher, USA) using the following steps: an initial denaturation at 94 °C for 5 min, 32 cycles of denaturation at 94 °C for 30 s, annealing at 55 °C for 30 s, and extension at 72 °C for 30 s, and finally an elongation step at 72 °C for 10 min. The amplified products with DL2000 DNA marker (Tecan, Switzerland) were electrophoresed on 1.5 % agarose gels which were stained with Cell Red Nucleic acid dye solution (Shanghai Yisheng Biotechnology Co., LTD.) and a voltage of 120 V for 1 h. After electrophoresis, photographs were taken in GelDoc™ XR + gel imager (Bio-Rad Corporation).

### 2.3 Sample preparation

**Fig. 2:**
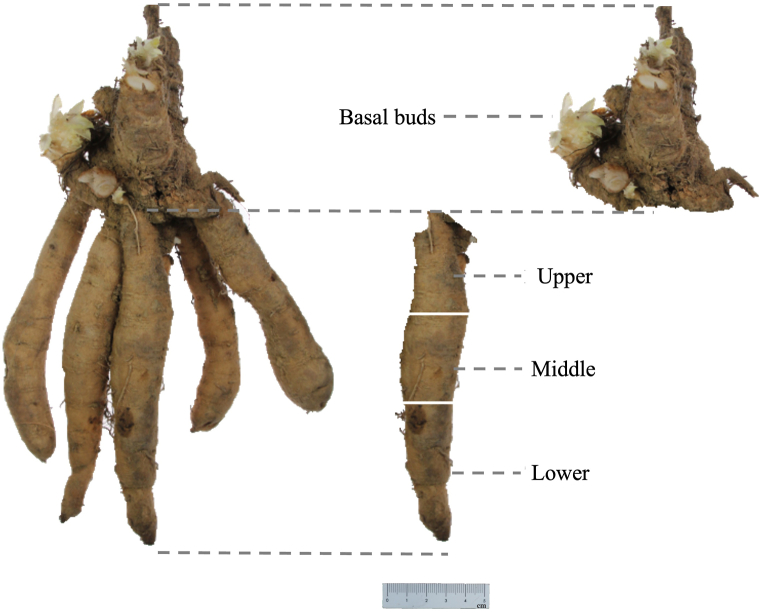
Underground parts of *S. ningpoensis*. Basal buds: originate at the base of *S. ningpoensis*, serve as reserves for new growth, in this study, basal buds part refers to the part where the stem connects to the root. The root of *S. ningpoensis* was segmented into three equal sections based on the length: upper part, middle part, lower part.

All the samples we analyzed in this experiment were manually harvested under the same method in the same day. After harvesting, all samples were cleaned and uniformly sliced. Subsequently, all the samples were placed simultaneously in an electric thermostatic drying oven (Shanghai Yiheng Scientific Instrument Co., Ltd) at 60 °C for 24 h to remove moisture. And the dried samples were pulverized using analytical mill (Germany, IKA A11 Basic), then filtered through a No. 3 sieve as per the specifications outlined in the Chinese Pharmacopoeia to obtain the dried powdered sample. And four levels of experiments were designed for the quality analysis. Level one: three robust individuals from each cultivated variety of *S. ningpoensis* were randomly selected, and from each individual plant, three well-developed roots were chosen and processed into dried powder, which was subsequently mixed, and the obtained mixed powder for each cultivated variety of *S. ningpoensis* was used for the quality comparison of nine different cultivated varieties. Additionally, the entire underground part of *S. ningpoensis* was divided into four parts: basal buds part, upper part, middle part, and lower part (Fig. 2). The basal buds part, upper, middle, and lower section of one well-developed root from each of the nine cultivated varieties were assessed for quality comparison of different parts of the same root. Level two: twenty different roots of *S. ningpoensis* from DP variety, which was considered a good quality variety [28], was processed for chemical variation analysis of different medicinal materials from the same variety. Level three: twelve different medicinal materials derived from the middle part of the roots of *S. ningpoensis* from DP variety was processed for the analysis of the chemical variation of controlling the spatial part of the roots. Level four: six different medicinal materials derived from the middle part of the roots under 20 g was processed for the analysis of controlling the mass of the medicinal materials. For possible application of future production, we chose the middle part of the root of *S. ningpoensi*s which is normally the part possessed with most of the mass to analyze the chemical variation compared to the whole roots.

0.5 g of dried sample powder was soaked in 50 mL of 50 % methanol aqueous solution (v/v) for 1 h in a stoppered 100 mL Erlenmeyer flask. The solution was sonicated at room temperature for 45 min, followed by filtering through a 0.45 μm syringe filter. The filtered solution was analyzed by HPLC within 24 h.

### 2.4 Quantitative analysis of chemical composition in Radix Scrophulariae

The chemical composition in Radix *Scrophulariae* was quantitively analyzed on an Agilent 1260 HPLC at 30 °C, equipped with an Agilent ZORBAX Eclipse XDB-C18 (4.6 × 250 mm, 5 μm) column and Agilent 1260 VWD detection (Agilent, USA). The mobile phase was composed of acetonitrile (A) and 0.02 % phosphoric acid aqueous solution (B), applied with the following gradients: 0–20 min, 3–5% A; 20–35 min, 5–15 % A; 35–55 min, 15–23 % A; 55–75 min, 23–65 % A; 75–77 min, 65–80 % A; 77–82 min, 80 % A; 82–84 min, 80-3% A. The flow rate was 1 mL/min, and the injection volume was 10 μL. The content of the chemical composition was determined by measuring the sample absorbance at 210 nm and draw standard curves as follows: aucubin: y = 265.13 x + 3.1728 (R^2^ = 0.9999, 0.127–3.171 μg); harpagide: y = 265.13 x + 3.1728 (R^2^ = 0.9999, 0.127–3.171 μg); acteoside: y = 1204.7 x + 2.0309 (R^2^ = 1, 0.020–0.800 μg); angoroside C: y = 4651.4 × - 2.1016 (R^2^ = 1, 0.010–0.400 μg); harpagoside: y = 1642.4 × - 0.4939, (R^2^ = 1, 0.037–0.905 μg); cinnamic acid: y = 5611.9 × - 2.0152, (R^2^ = 1, 0.006–0.231 μg).

### 2.5 Data analysis

Based on the electrophoretogram, the bands with clear and repeatable image were counted. We designated amplified fragments of markers as 0 in the absence of a band and 1 in the presence. POPGENE 32.0 software was used to calculate the genetic parameters of varieties, including percentage of polymorphic bands (PPB), observed number of alleles (N_a_) and effective number of alleles (N_e_), Nei's gene diversity index (H), Shannon's Information index (*I*), total gene diversity (H_t_), gene diversity within populations (H_s_), Nei's coefficient of gene differentiation (Gst), the estimate of gene flow from Gst (N_m_) [27]. Unweighted Pair-group Method Analysis (UPGMA) was used to perform cluster analysis on samples and plot tree graphs was generated using NTSYS-pc 2.10e software [29]) and online website (https://itol.embl.de/). GenAIEx 6.5 was used carry out Principal Coordinate Analysis (PCoA) [30].

To analyze the quality variation of Radix *Scrophulariae*, we used GraphPad prism 8 software to draw the bar chart [31]. Chromatographic Fingerprint of TCM (Version 2012) was used to draw the fingerprints of Radix *Scrophulariae* and identify the characteristic peak. Using online website (https://www.chiplot.online/) for clustering heat map, principal component analysis (PCA) and correlation analysis graphs. IBM SPSS Statistics 26.0 software was used to conduct Duncan multiple comparison [32], the Euclidean distance calculation based on standardized 18 HPLC characteristic peak areas [33], Pearson correlation analysis among 6 ingredients [34]. According to the formula of coefficient of variation: *C.V* = *SD/MN* * 100 % (*C.V*: coefficient of variation; *SD*: standard deviation; *MN*: average value) to calculate the quality variation of Radix *Scrophulariae* [35].

To clarify the correlation between genetic differentiation and chemical variation of Radix *Scrophulariae*, we compared the genetic distance matrix of different varieties with the Euclidean distance matrix of 18 HPLC characteristic peak areas and the content of 6 ingredients of the whole underground part of *Scrophularia ningpoensis* Hemsl. By performing a 999 permutation Pearson correlation coefficient calculation using the vegan Mantel function in R to assess how well the chemical composition matched the genetic background [36]. Additionally, to explore the potential of the middle part of the root of *Scrophularia ningpoensis* Hemsl. As a premium medicinal herb, the correlation between genetic differentiation and chemical variation of the middle part of the root of *Scrophularia ningpoensis* Hemsl. Was analyzed compared to the whole roots.

## 3 Results

### 3.1 Genetic differentiation of S. ningpoensis from different varieties

#### 3.1.1 Polymorphism analysis of amplification products

The 13 SRAP primer sets (Table 2) utilized to examine genetic variation of *S. ningpoensis* generated a total of 4–14 bands with an average of 8.85 bands per primer combination. A total of 115 bands were scored, of which 103 (90.43 %) were polymorphic and 12 (9.57 %) were monomorphic. There were 3 primer sets with 100 % polymorphism, accounting for 23.08 % of the total primers sets. Approximately 2–6 bands, with an average of 2.67 bands per primer set, were produced with the 12 SSR primer combinations (Table 2). A total of 32 bands were scored, of which 20 (62.50 %) were polymorphic and 12 were monomorphic through all genotype tested. There were 2 pairs of primer sets with 100 % polymorphism, accounting for 16.67 % of the total primer sets. The primer sequences were displayed in Table S1.

**Table 2:** The polymorphic information of amplified bands obtained by SSR and SRAP markers.

Marker Type	Marker name	TNB	NPB	PPB (%)	N_a_	N_e_	H	*I*
SRAP	Me4-Em7	7	5	85.71	1.8571	1.4724	0.284	0.4324
	Me3-Em5	8	8	100.00	2.0000	1.4367	0.2751	0.4341
	Me3-Em16	9	8	88.89	1.8889	1.3782	0.2419	0.3832
	Me1-Em8	8	7	87.50	1.8750	1.4003	0.2426	0.3793
	Me3-Em15	8	8	100.00	2.0000	1.4728	0.2959	0.4605
	Me13-Em10	4	3	75.00	1.7500	1.5702	0.3195	0.4619
	Me4-Em5	14	13	92.86	1.9286	1.5018	0.3009	0.4583
	Me7-Em16	10	9	90.00	1.9000	1.5650	0.3361	0.4999
	Me3-Em10	10	9	90.00	1.9000	1.5053	0.3030	0.4590
	Me6-Em9	12	11	91.67	1.9167	1.5895	0.3491	0.5186
	Me2-Em4	6	5	83.33	1.8333	1.4743	0.2761	0.4184
	Me9-Em6	8	6	75.00	1.7500	1.2879	0.1834	0.2960
	Me8-Em8	11	11	100.00	2.0000	1.6752	0.3873	0.5699
	Average	8.46	7.54	90.43	1.9043	1.4943	0.2968	0.4512
SSR	SSR-1	2	1	50.00	1.5000	1.1760	0.1302	0.2147
	SSR-2	3	2	66.67	1.6667	1.5129	0.2840	0.4101
	SSR-3	6	4	66.67	1.6667	1.3909	0.2367	0.3570
	SSR-4	2	1	50.00	1.5000	1.2752	0.1775	0.2701
	SSR-5	2	1	50.00	1.5000	1.1760	0.1302	0.2147
	SSR-6	4	4	100.00	2.0000	1.9044	0.4734	0.6660
	SSR-7	2	1	50.00	1.5000	1.4941	0.2485	0.3451
	SSR-8	2	1	50.00	1.6667	1.3548	0.2051	0.3125
	SSR-9	2	1	50.00	2.0000	1.4512	0.3077	0.4848
	SSR-10	2	1	50.00	1.6250	1.4064	0.2359	0.3497
	SSR-11	2	1	50.00	1.5000	1.2752	0.1775	0.2701
	SSR-12	3	1	33.33	1.3333	1.2474	0.1420	0.2057
	Average	2.67	1.54	62.50	1.6250	1.4064	0.2359	0.3497

Footnote: TNB: total number of bands, NPB:number of polymorphic bands, PPB: the percentage of polymorphic bands, N_a_: the observed number of alleles, N_e_: the effective number of alleles, H: Nei's gene diversity index, *I*: Shannon's Information index.

#### 3.1.2 Results of genetic diversity

The results of genetic diversity based on SRAP markers showed that at the species level, the N_a_ and N_e_ were 1.9043 and 1.4943, H was 0.2968, *I* was 0.4512, PPB was 90.43 %. All of them were higher than that of the varieties from Zhejiang origins (N_a_: 1.6000, N_e_: 1.3669, H: 0.2153, *I*: 0.3221, PPB: 60.00 %) and the varieties from southwest China origins (N_a_: 1.6087, N_e_: 1.3692, H: 0.2112, *I*: 0.3151, PPB: 60.87 %). As for SSR markers, the results were identical to SRAP markers. At the species level, the N_a_ and N_e_ were 1.6250 and 1.4046, H was 0.2359, *I* was 0.3497, PPB was 62.50 %. It also showed better genetic diversity than that of the varieties from Zhejiang origins (N_a_: 1.4375, N_e_: 1.3669, H: 0.2153, *I*: 0.3221, PPB: 60.00 %) and varieties from southwest China origins (N_a_: 1.6087, N_e_: 1.3692, H: 0.2112, *I*: 0.3151, PPB: 60.87 %). For the results of both markers showed consistency, it proved that the genetic diversity of *S. ningpoensis* from same origin was smaller than that from different origins. The results of SRAP markers showed that between the varieties in Zhejiang and southwest China, H_t_ was 0.2733, H_s_ was 0.2132, Gst was 0.2198, and N_m_ was 1.7750. And the results of SSR markers was as follows: H_t_: 0.2508, H_s_: 0.1596, Gst: 0.3638, and N_m_: 0.8743 (Table 3).

**Table 3:** The genetic diversity parameters of *S. ningpoensis* in two main production areas in China.

	Origins	N_a_	N_e_	H	*I*	PPB%	H_t_	H_s_	Gst	N_m_
SRAP	Zhejiang	1.6000	1.3669	0.2153	0.3221	60.00				
	Southwest	1.6087	1.3692	0.2112	0.3151	60.87				
	Average	1.6044	1.3681	0.2133	0.3186	60.44	0.2733	0.2132	0.2198	1.7750
SSR	Zhejiang	1.4375	1.2675	0.1600	0.2395	43.75				
	Southwest	1.4062	1.2786	0.1592	0.2339	40.62				
	Average	1.4219	1.2731	0.1596	0.2367	42.19	0.2508	0.1596	0.3638	0.8743

Footnote: N_a_: the observed number of alleles, N_e_: the effective number of alleles, H: Nei's gene diversity index, *I*: Shannon's information index, PPB: the percentage of polymorphic bands, H_t_: total gene diversity, H_s_: gene diversity within populations, Gst: Nei's coefficient of gene differentiation, N_m_: the estimate gene flow of Gst.

#### 3.1.3 Genetic distance analysis

The genetic distance between DP and FQ from Zhejiang origins based on SRAP markers was 0.0445. The same coefficient reached to 0.3629–0.4823 when the cultivated and wild *S. ningpoensis* from Zhejiang origins were compared. Among the varieties of southwest China, the genetic distance was 0.0628–0.4545. There was a trend of regionalization of genetic distance when it came to the comparison of all *S. ningpoensis* we collected. The genetic distance between Zhejiang and southwest China origins increased to 0.3882–0.6178. The results of genetic distance based on SSR markers were identical to those of SRAP markers (Table S2).

#### 3.1.4 Cluster analysis based on UPGMA and PCoA

**Fig. 3:**
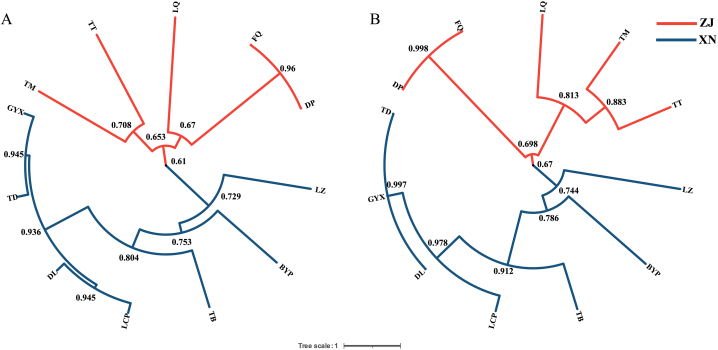
Dendrogram based on UPGMA of *S. ningpoensis* of different varieties. A: based on the genetic similarity coefficient from the matrix data of electrophoresis bands from SRAP markers. B: based on the genetic similarity coefficient from the matrix data of electrophoresis bands from SSR markers. The red line represents varieties from Zhejiang, the blue line represents varieties from southwest China. ZJ: Zhejiang group, TM, TT, LQ, FQ, DP are varieties from Zhejiang; XN: southwest China group, LZ, BYP, TB, LCP. DL, TD, GYX are varieties from southwest China. The cluster trees are generated by NTSYS-pc 2.10e software and modified at online website (https://itol.embl.de/). (For interpretation of the references to color in this figure legend, the reader is referred to the Web version of this article.)

The genetic similarity coefficient among different varieties ranged from 0.61 to 0.95 (SRAP) and 0.67–0.998 (SSR). As shown in Fig. 3A, at the genetic similarity coefficient of 0.653 (SRAP), DP, FQ, LQ, TM and TT from Zhejiang were clustered into one group. TB, TD, GYX, DL, LCP, BYP and LZ from southwest China were clustered into another group at the genetic similarity coefficient of 0.668 (SRAP). The dendrogram based on SSR markers showed similar results (Fig. 3B). The results indicated that there were obvious genetic differences between the two production areas.

**Fig. 4:**
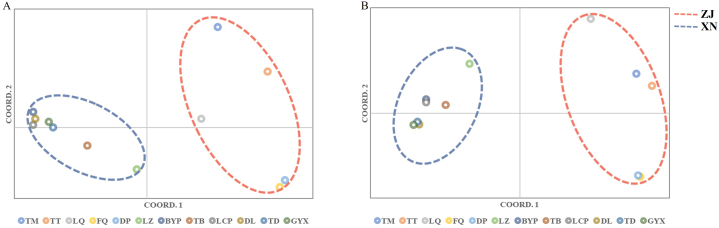
Scatter plots of Principal Coordinate Analysis (PCoA) of *S. ningpoensis* of different varieties. A: PCoA graphic result based on genetic distance from the matrix data of electrophoresis bands from SRAP markers, B: PCoA graphic result based on genetic distance from the matrix data of electrophoresis bands from SSR markers. The dots with different color represent different varieties of *S. ninggpoensis*. The dots circled by red dashed line represent varieties from Zhejiang, the dots circled by blue dashed line represent varieties from south west China. ZJ: Zhejiang group, TM, TT, LQ, FQ, DP are varieties from Zhejiang; XN: southwest China group, LZ, BYP, TB, LCP, DL, TD, GYX are varieties from southwest China. The PCoA graphics were generated by GenAIEx 6.5 Excel 2021 plugin. (For interpretation of the references to color in this figure legend, the reader is referred to the Web version of this article.)

The two principal coordinates based on PCoA accounted for 50.27 % (SRAP) and 64.80 % (SSR) respectively (Fig. 4A and B). The 12 varieties of *S. ningpoensis* were mainly divided into two groups. The first group contained the varieties of TB, TD, GYX, DL, LCP, BYP, and LZ from southwest China. The second group was consisted by the varieties of TM, TT, LQ, DP, FQ from Zhejiang.

### 3.2 Quality evaluation of Radix Scrophulariae

#### 3.2.1 HPLC fingerprints construction

**Fig. 5:**
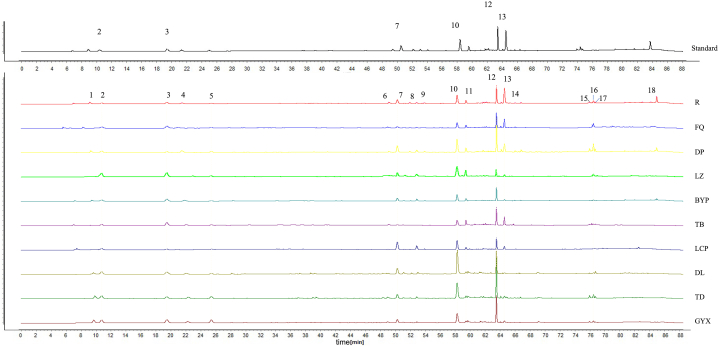
HPLC fingerprints of 9 cultivated varieties of Radix *Scrophulariae*. Eighteen HPLC peaks were detected, six selected standard substances are as follows: (2: Aucubin, 3: Harpagide, 7: Acteoside, 10: Angoroside C, 12: Harpagoside, 13: Cinnamic acid).

The samples’ solution showed good signal sensitivity at 210 nm wavelength, and a total of 18 HPLC characteristic peaks were counted within 90 min. Compared with the chromatogram of standard substances, peaks 2, 3, 7, 10, 12 and 13 were identified as aucubin, harpagide, acteoside, angoroside C, harpagoside and cinnamic acid (The chromatogram of all standards with details was shown in Fig. S2). Their retention times were 10.460, 18.985, 48.391, 56.532, 62.661 and 63.568 min, respectively (Fig. 5).

#### 3.2.2 The chemical variation of Radix Scrophulariae from different producing area

**Fig. 6:**
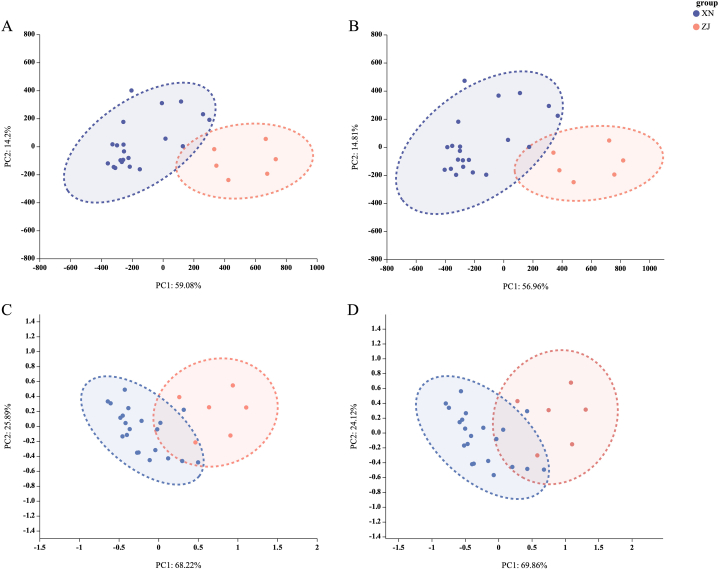
Principal component analysis of 9 cultivated Radix *Scrophulariae.* A: based on 18 HPLC characteristic peak areas of whole underground part, B: based on 18 HPLC characteristic peak areas of middle roots. C: based on the content of six ingredients of whole underground part, D: based on the content of six ingredients of middle roots. Points circled by red dashed line are varieties from Zhejiang, points circled by blue dashed line are varieties from southwest China. (ZJ: Zhejiang, XN: southwest China). (For interpretation of the references to color in this figure legend, the reader is referred to the Web version of this article.)

The PCA results of chemical composition of Radix *Scrophulariae* from Zhejiang and southwest China were clearly divided into two groups as shown in Fig. 6. The total explanation rate was 71.77 %–94.11 % in the four sets of PCA analysis. According to both 18 HPLC characteristic peak areas (Table S3) and 6 ingredients' data sets (Table S4), we found that the total explanation rate of the PCA analysis based on data sets of the whole underground part (Fig. 6A and C; 73.28 %, 94.11 %) was slightly higher than that of the middle roots (Fig. 6B and D; 71.77 %, 93.98 %). Compared the score's plots based on 18 HPLC characteristic peaks (Fig. 6A and B) with the score's plots based on six ingredients (Fig. 6C and D) in the four graphs, we found that the 18 HPLC characteristic peak areas better reflected the differentiation of chemical composition between Radix *Scrophulariae* from Zhejiang and southwest China than the content of six ingredients.

**Fig. 7:**
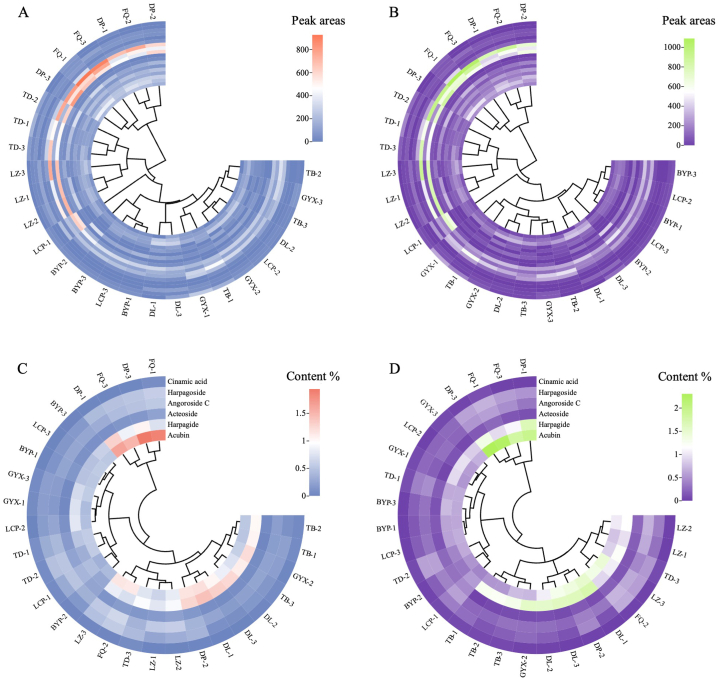
The clustering heatmaps of nine different cultivated varieties. A, B: based on the 18 HPLC characteristic peak areas; C, D: based on the content of the 6 ingredients. A, C: chemical composition derived from the whole underground part; B, D: chemical composition derived from the middle part of the roots. Each sample was replicated three times.

The clustering heatmaps based on the chemical composition of Radix *Scrophulariae* showed that the different varieties from Zhejiang and southwest China were also divided into two categories (Fig. 7). Clustering heatmaps based on 18 HPLC characteristic peak areas (Fig. 7A and B) were more accurate than that based on the content of six ingredients (Fig. 7C and D). The chemical composition of Radix *Scrophulariae* from Zhejiang and southwest China were clustered into their own group respectively in the clustering heatmap of HPLC characteristic peaks (Fig. 7A and B), while two varieties originated from Zhejiang were categorized into the group of southwest China in the heatmap based on the content of six ingredients (Fig. 7C and D). The results indicated that chemical composition varied between the varieties from Zhejiang and southwest China, HPLC characteristic peaks could better reflect the chemical variation among the different varieties originated from different places, and instead of middle roots, the whole underground part could better explain the quality difference of Radix *Scrophulariae.*

#### 3.2.3 Correlation between genetic differentiation and chemical variation of S. ningpoensis

To explore the correlation between the genetic differences and the differences in chemical composition of *S. ningpoensis*, we conducted a correlation analysis between the genetic distance matrix based on SRAP and SSR molecular markers and the Euclidean distance matrix of chemical composition, as shown in Table 4. It can be observed from the table that there is a significant correlation between the genetic distance and the Euclidean distance matrix of *S. ningpoensis*. The better correlation of genetic distance matrix based on SRAP compared to SSR might be attributed to the richer band information of SRAP molecular markers. The higher correlation of the whole roots of *S. ningpoensis* compared to the middle part of the roots might indicate that the whole roots better reflect the chemical differences among different varieties. The better correlation of 18 characteristic peak areas compared to the content of 6 ingredients suggested that a greater number of chemical components better represent the chemical differences among different varieties of *S. ningpoensis.* These findings indicate that substantial differences exist between the whole root and the middle part of the root when the analysis targets a limited number of components. Conversely, when the analysis encompasses multiple components, the distinctions between the whole root and the middle part of the root are less pronounced.

**Table 4:** The Pearson correlation analysis of genetic distance matrix and Euclidean distance matrix.

HPLC data Molecular data	Matrix A	Matrix B	Matrix C	Matrix D
Matrix-SRAP	r = 0.7196, p = 0.011	r = 0.713, p = 0.008	r = 0.6239, p = 0.004	r = 0.3996, p = 0.03
Matrix-SSR	r = 0.6559, p = 0.007	r = 0.6303, p = 0.01	r = 0.5821, p = 0.013	r = 0.4063, p = 0.036

Footnote: matrix A, B correspondingly represents Euclidean distance matrix based on 18 characteristic peak areas from the whole roots (Table S5) and the middle part of the roots (Table S6); matrix C, D correspondingly represents Euclidean distance matrix based on the content of 6 ingredients from the whole roots (Table S7) and the middle part of the roots (Table S8); matrix SRAP, SSR correspondingly represents the genetic distance matrix based on the matrix data of electrophoresis bands from SRAP markers and SSR markers (Table S2).

#### 3.2.4 Association between single ingredient and genetic variation

To investigate the correlation between the content of 6 components and genetic variations, we separately conducted Pearson correlation analysis on the Euclidean distance matrix of each of the content of 6 components based on the whole roots (Table S9) and the middle part of the roots (Table S10) and the genetic distance matrix based on SRAP and SSR markers (Table S2). In the correlation results (Table 5) between the Euclidean distance matrix of individual component in Radix *Scrophulariae* and the genetic distance matrix of *S. ningpoensis*, there was a significant positive correlation with aucubin. The correlation based on SRAP markers was stronger than that of SSR markers might be due to the higher polymorphism information content of SRAP markers. Similarly, the stronger correlation of the Euclidean distance matrix based on the whole roots of *S. ningpoensis* indicates that the whole roots better reflect the differences among different varieties when it comes to a limited number of components. This result suggests the potential of aucubin as a germplasm chemical marker for *S. ningpoensis*.

**Table 5:** The correlation coefficient between the content of each active ingredient and the genetic difference of *S. ningpoensis* from different varieties.

HPLC data Molecular data	Aucubin-W	Harpagide-W	Acteoside-W	Angoroside *C*–W	Harpagoside-W	Cinnamic acid-W
Matrix-SRAP	0.7114**	0.2882	0.1126	0.2023	0.3337	−0.1034
Matrix-SSR	0.608*	0.2246	0.2012	0.2085	0.2743	−0.1018
HPLC data Molecular data	Aucubin-M	Harpagide-M	Acteoside-M	Angoroside *C*-M	Harpagoside-M	Cinnamic acid-M
Matrix-SRAP	0.5586*	−0.08472	0.0725	0.1262	0.2864	−0.08671
Matrix-SSR	0.4686*	−0.1642	0.1322	0.1333	0.2237	−0.08582

Footnote: **: p < 0.01, *: p < 0.05; W: the Euclidean distance matrix based on the HPLC data from the whole roots (Table S9); M: the Euclidean distance matrix based on the HPLC data from the middle part pf the roots (Table S10).

#### 3.2.5 The coefficient of variation of chemical composition of Radix Scrophulariae

**Fig. 8:**
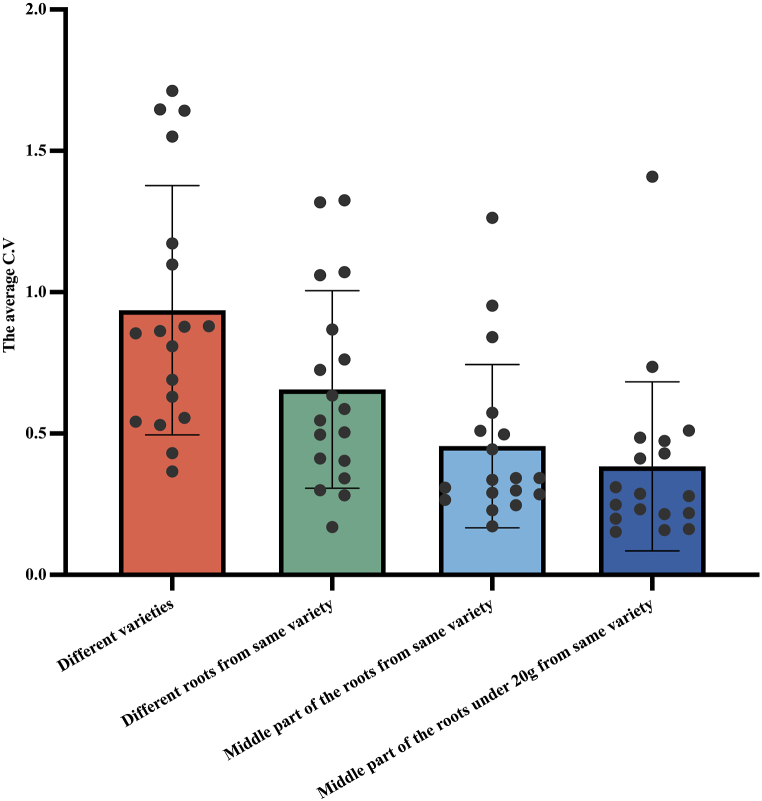
Coefficient of variation (*C.V*) of chemical composition of different Radix *Scophulariae*. Dots represent the *C.V* of 18 characteristic peaks, C.V was calculated by 18 characteristic peak areas. Different varieties: all medicinal materials derived from the underground part of *S. ningpoensis* from 9 cultivated varieties (Table S3); different roots from same variety: 20 medicinal materials derived from DP variety (Table S11); middle part of the roots from same variety: medicinal materials derived from the middle part of the roots of *S. ningpoensis* from DP variety (Table S12); middle part of the roots under 20 g from same variety: medicinal materials derived from the middle part of the roots under 20 g from DP variety (Table S12).

In the analysis of *S. ningpoensis* involving HPLC and its correlation with genetics, it is evident that the utilization of 18 HPLC characteristic peaks relative to the content of 6 chemical components exhibit better discriminative capabilities for different varieties of *S. ningpoensis*. Consequently, we utilized the Coefficient of Variation (*C.V*) formula to calculate the chemical variation coefficient of different Radix *Scrophulariae* based on the areas of the 18 HPLC characteristic peaks. Four different levels of Radix *Scrophulariae* were analyzed: 1) all medicinal materials from different varieties; 2) Radix *Scrophulariae* derived from DP variety; 3) medicinal materials derived from the middle part of the roots of *S. ningpoensis* from DP variety; 4) medicinal materials derived from the middle part of the roots under 20 g from same variety of DP. The obtained results are presented in Fig. 8. The coefficient of variation of chemical composition of different materials of Radix *Scrophulariae* of 9 different cultivated varieties reached an average of 93.62 %; the coefficient of variation of chemical composition of different materials of Radix *Scrophulariae* from same variety DP reached an average of 64.21 %; the coefficient of variation of chemical composition of different materials of Radix *Scrophulairae* from the middle part of the roots of *S. ningpoensis* from DP reached an average of 45.55 %; the coefficient of variation of chemical composition of Radix *Scrophulariae* with same genetic background, derived from the same spatial part of the roots and approximate mass under 20 g can be reduced to 38.43 %.

#### 3.2.6 Correlation analysis between chemical composition of Radix Scrophulariae and prediction of synthetic pathway

**Fig. 9:**
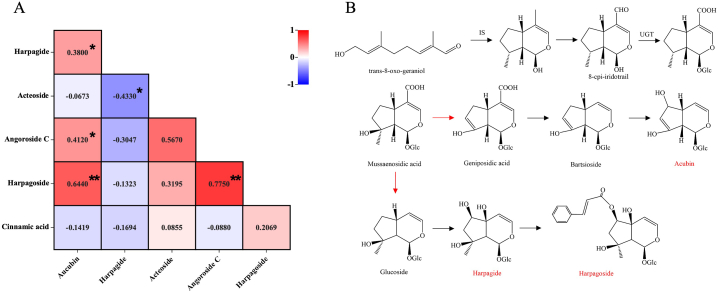
Correlation analysis of six ingredients of Radix *Scrophulariae* (A) and prediction of synthetic pathway (B) of the three iridoids (aucubin, harpagide and harpagoside).

The result of correlation analysis between each of the six ingredients (Fig. 9A) showed that there was a significant positive correlation between aucubin and harpagide, r = 0.5190 (P < 0.05); a significant positive correlation between aucubin and harpagide, r = 0.5310 (P < 0.05). Acteoside had significant positive correlations with angoroside C and harpagoside, r = 0.7250 (P < 0.01) and r = 0.5090 (P < 0.01), respectively. Angoroside C had a significant positive correlation with harpagoside, with r = 0.8670 (P < 0.05).

Harpagide, harpagoside, and aucubin are all iridoids, and the synthesis pathway of iridoids in Radix *Scrophulariae* is mainly through the cyclization reaction of *trans*-8-oxo-geraniol catalyzed by iridoid synthase, which leads to the formation of 8-cpi-iridotrail and further production of mussaenosidic acid. Mussaenosidic acid has two metabolic routes: one leads to the formation of glucosides and then to the synthesis of harpagide, which is further converted to harpagoside. The other leads to the formation of geniposidic acid and then to the synthesis of aucubin [[37], [38], [39]] (Fig. 9B). In this pathway, aucubin shows a significant positive correlation with harpagoside (r = 0.5190, P < 0.05) and harpagide (r = 0.5310, P < 0.05), possibly because all three compounds are derived from the same precursor compound, mussaenosidic acid. When the biomass of mussaenosidic acid increases, the biosynthesis of aucubin, harpagide, and harpagoside also increases.

## 4 Discussion

### 4.1 The impact of genetic differentiation on the quality of medicinal plants

Sexual reproduction and agamic propagation are two main modes of reproduction of medicinal plants. Medicinal plants propagated by seed such as the Indian ginseng *Withania somnifera* (L.) Dunal not only possessed with great genetic diversity, but also differed significantly in the content of aferin and anolide A within one population [40]. The genetic background of asexually propagated medicinal plants from the same origin is relatively homogeneous. Thus, for *S. ningpoensis* was produced asexually, we did not regard different individuals as the variables with great influence when discussing the different mass of the roots of *S. ningpoensis* from the same variety. But the genetic differences of different medicinal plants in different producing areas are significant which lead to chemical variation. Tahir et al. [19] found that *maize* from three producing regions of Iraq showed significant positive correlation between the genetic differences and chemical variation (r = 0.21, p = 0.0001). Research of *African prunus* also showed that the genetic distance matrix based on chloroplasts and nuclei were positively correlated with both the concentration of ursolic acid in bark and the distance matrix composed of all chemical components in bark [20]. Research of *Scrophularia ningpoensis* Hemsl. From Chen et al. [41] showed that after hundreds of years’ domestication, *S. ningpoensis* has experienced dramatic loss of genetic diversity, the genetic differentiation between cultivated populations and wild populations was significant, three cultivated populations from Zhejiang grouped together in principal coordinate analysis, it indicated that the advantage in chemical variation of populations of *S. ningpoensis* from Zhejiang might partly be due to the genetic differentiation. And the research of Yang et al. [21] showed that between the cultivated and wild populations of *S. ningpoensis*, the genetic differentiation was parallel to chemical variation.

In this study, the genetic differences among 9 cultivated varieties and 3 wild accessions of *S. ningpoensis* were clearly clarified by the analysis of genetic parameters of the observed number of alleles (N_a_), effective number of alleles (N_e_), Shannon's Information index (*I*), Nei's gene diversity (H) as well as Nei's genetic distance, which were of great significance for the genetic analysis of medicinal plants*.* The gene diversity analysis based on SRAP and SSR markers (Table 2) both showed abundant genetic diversity which reflected the good environmental adaptability and viability of *S. ningpoensis* [42]. The varieties from Zhejiang had obvious genetic differentiation compared to those from southwest China. In the meantime, the chemical composition of varieties from Zhejiang was also significantly varied from southwest China. And based on Mantel tests which the genetic and chemical variation of *S. ningpoensis* were highly correlated, we inferred that the chemical variation of Radix *Scrophulariae* from different varieties were largely affected by the genetic differentiation. The closer the genetic distance, the more similar the chemical composition. This study utilized SRAP and SSR molecular marker techniques. The synergistic application of these two marker types was instrumental, with SSR markers exhibiting abundant coverage across the entire genome, while SRAP markers are relatively more stable. In this study, it was observed that SRAP markers provide more polymorphic information compared to SSR markers, and the result of SRAP markers was basically consistent with the research conducted by Chen et al. [11] Additionally, the genetic analysis results of SRAP and SSR markers in this study were largely consistent, indicating a certain level of reliability in the obtained results.

### 4.2 The positive significance of chemical variation control for pharmacodynamic stability

Due to the long environmental openness of the production model, there are many factors affecting the efficacy of traditional Chinese medicine, including its origin and processing methods [[43], [44], [45]]. But it is essentially difference in the content of the medicinal ingredients that may cause the different pharmacodynamic efficacy. The chemical composition of medicinal plants varies not only from different origins but also from different parts of the medicinal materials. Such as *Ginkgo Biloba* L [46]. and *Hypericum montbretti* Spach [47]. exhibited chemical variation across different origins and the content of volatile oil of different parts of the *Angelica sinensis* also significantly showed significant chemical variation [48]. Xie et al. [49] studied different parts of *S. ningpoensis* including roots, stem and leaves, and found that the stem could possess high content of aucubin (30.09 mg/g) and harpagide (28.4 mg/g) in August and the content of harpagoside in the leaves could reach 12.02 mg/g in July, but the different parts of the underground part of *S. ningposnsis* was not included for discussion.

This study investigated the chemical composition of *S. ningpoensis* from different geographical origins and examined the correlation between its genetics and chemical components using two sets of data: eighteen HPLC chromatographic peaks and the content of six specified components. We observed that the dataset comprising eighteen chromatographic peaks better discriminated between *S. ningpoensis* samples from different geographical origins in PCA and cluster heatmaps. Moreover, in the analysis of the correlation between genetics and chemical components, the dataset of eighteen chromatographic peaks exhibited superior correlations compared to the dataset of six component contents. Furthermore, we compared the whole underground part of *S. ningpoensis* with its middle part of the root. Both the whole underground part and the intermediate section effectively distinguished *S. ningpoensis* from the southwest China and Zhejiang regions in PCA and cluster heatmap analyses. In the correlation analysis between genetics and chemical components, it can be observed that the HPLC data set of 18 peaks of whole underground part was slightly more related to genetics than the middle part of the roots; but when refers to 6 chemical components, the data set based on the whole underground part was significantly more related to genetics than the middle part of the roots (Table 4). This result illustrated that when multiple components are chosen as the determination targets, minimal distinctions were observed between the whole roots and the underground part and the middle part of the root, however, when a minority of chemical components are selected for analysis, the whole underground part proves to be more indicative to the differences among different varieties.

This study investigated the coefficient of variation (*C.V*) of chemical composition of Radix *Scrophulariae* based on 18 HPLC peaks with different levels: 1) Radix *Scrophulariae* from different varieties; 2) Radix *Scrophulariae* from the same variety DP; 3) Radix *Scrophulariae* from the middle part of the roots of *S. ningpoensis* from same variety of DP; 4) Radix *Scrophulariae* from the middle part of the roots under 20 g from same variety of DP. The *C.V* of chemical composition of Radix *Scrophulariae* of 9 different cultivated varieties reached an average of 93.62 %; the *C.V* of chemical composition of Radix *Scrophulariae* from the same variety DP reached an average of 64.21 %; the *C.V* of chemical composition of Radix *Scrophulairae* from the middle part of the roots of *S. ningpoensis* from DP reached an average of 45.55 %; the *C.V* of chemical composition of Radix *Scrophulariae* with same genetic background, from the middle part of the roots with approximate mass under 20 g was 38.43 %. And this result illustrated that the genetic background, different spatial part of the roots, different development of roots were three non-environmental factors causing the chemical variation of Radix *Scrophulariae*, and we could reduce the chemical variation of Radix *Scrophulariae* during production by selecting medicinal materials with approximate mass under 20 g derived from the same part of the roots from the same variety of the medicinal plants.

The correlation of single ingredient with genetics suggested the potential of aucubin as a germplasm chemical marker for *S. ningpoensis* (Table 5). Previous studies showed that there might be metabolic relationships between aucubin, harpagide and harpagoside [37], the results of the correlation analysis on six components in Radix *Scrophulariae* showed that aucubin was significantly positively correlated with harpagide and harpagoside. This result could be due to their sharing an upstream compound mussaenosidic acid, and the pathway of the metabolism needs further research.

## 5 Conclusion

Traditional Chinese medicine has a long history of clinical application and is an important part of Chinese medical system. Chinese medicinal materials have the characteristics of complex chemical components, which is the basis of the pharmacodynamic efficacy of Chinese medicine. However, the chemical composition of the medicinal materials is not as highly accurate as chemical drugs because of the highly open production norm. Thus, the considerable variation in its chemical composition affects clinical medication dosage judgment at a certain extent. There was obvious genetic differentiation in the two main producing areas of *S*. *ningpoensis*, and the coefficient of genetic distance reached above 0.3882. This study showed that genetic factors were the main non-environmental factors affecting the chemical variation of Radix *Scrophulariae* from nine cultivated varieties, which caused the coefficient of variation to reach 93.62 % even with the same cultivating environment. The coefficient of variation of chemical composition of Radix *Scrophulairae* from the same variety reached 64.21 %. The coefficient of variation of chemical composition of Radix *Scrophulariae* derived from the middle part of the roots of the same variety DP reached 45.55 %. By controlling genetic background, processing uniformity, spatial part and plant organ size of the medicinal materials, the chemical variation coefficient of Radix *Scrophulariae* can be significantly reduced to 38.43 %. The production and application of Chinese herbal medicines with low coefficient of variation will be conducive to the standardization of downstream Chinese herbal medicine industry. The coefficient of correlation between the content of aucubin and the genetic differences of *S. ningpoensis* was significant (SRAP-W: 0.7114**; SSR-W: 0.608*; SRAP-M: 0.5586*; SSR-M: 0.4686*) illustrated that aucubin had the potential to be one of the chemical markers of *S. ningpoensis*. The composition of traditional Chinese medicinal materials is intricately complex. In this study, HPLC can only detect part of the chemical components, further in-depth research is needed on the additional medicinal substances, different parts of the roots and metabolic pathways of *S. ningpoensis*.

## Data availability statement

The original contributions presented in the study are included in the article/Supplementary Material, further inquiries can be directed to the corresponding author.

## CRediT authorship contribution statement

**Hui Yao:** Writing – review & editing, Writing – original draft, Visualization, Validation, Software, Methodology, Investigation, Formal analysis, Data curation. **Jian Sun:** Writing – review & editing, Supervision, Resources, Project administration, Methodology, Investigation, Funding acquisition, Formal analysis, Data curation, Conceptualization. **Mengying Chen:** Writing – review & editing. **Yu Dong:** Writing – review & editing. **Pan Wang:** Writing – review & editing. **Jianzhong Xu:** Writing – review & editing. **Qingsong Shao:** Writing – review & editing. **Zhian Wang:** Writing – review & editing, Supervision, Resources, Project administration, Investigation, Conceptualization.

## Declaration of competing interest

The authors declare that they have no known competing financial interests or personal relationships that could have appeared to influence the work reported in this paper.
