# Supplementary material for: The impact of non-environmental factors on the chemical variation of Radix Scrophulariae
Source: Heliyon. 2024 Jan 12;10(2):e24468. doi: 10.1016/j.heliyon.2024.e24468 (PMC10831622; doi:10.1016/j.heliyon.2024.e24468)
Supplement: Multimedia component 1 [file mmc1.docx]

Table S1 The sequences of SRAP and SSR markers.

| Primer name | Forward primer | Reverse primer |
| --- | --- | --- |
| Me4-Em7 | BACC | DATG |
| Me3-Em5 | BAAT | DAAC |
| Me3-Em16 | BAAT | DCGG |
| Me1-Em8 | BATA | DAGC |
| Me3-Em15 | BAAT | DCTG |
| Me13-Em10 | BCAG | DTAG |
| Me4-Em5 | BACC | DAAC |
| Me7-Em16 | BTTG | DCGG |
| Me3-Em10 | BAAT | DDTAG |
| Me6-Em9 | BTAG | DACG |
| Me2-Em4 | BAGC | DTGA |
| Me9-Em6 | BTCA | DGAC |
| Me8-Em8 | BTGT | DAGC |
| SSR1 | CAGTGGTAAATCTCCAGGCAT | CTTTGCTTTCTCTTTTATGGC |
| SSR2 | CTAAACCCCAAATCAACAACC | TCACCAAAGAGGGAACAAACT |
| SSR3 | TTACAAAGGTGAGTTTCGGTG | TACTCAACAAACCCGACAGAT |
| SSR4 | CGCAAAATCTGGACAACTATG | AGATTAGCGAAATGGGATACG |
| SSR5 | TCCTTGTGGAGGCTAATGACC | GCTATTCTCACCGCCAACATC |
| SSR6 | GGAGGAAGTGTTGACTAGCGG | ACTAGGTATTCGGTGCCCAAA |
| SSR7 | TCCAACATCCACACCTACACC | CCTTGCTGAATGACGAACACA |
| SSR8 | AATGAGTTCAAGGTGGAGGTG | GCACTGGTGATGTCTTCCTCT |
| SSR9 | AAAGTCAGTCCCCCAATAAAA | TTATCTCCCCCATCATCTTGT |
| SSR10 | TCTTTTTCCCCTTCATTTCTC | CCAGACATCTCACTATGCTTG |
| SSR11 | CTCTTTTCCAGGAGGGACAGT | CATCACCGCCATTATTACCAC |
| SSR12 | GATGAGGATGTTGTGGAGAATA | CTTTCTCTTCCTCACGCTCTT |

B = TGAGTCCAAACCGG, D=GACTGCGTACGAATT
