# Supplementary material for: The impact of non-environmental factors on the chemical variation of Radix Scrophulariae
Source: Heliyon. 2024 Jan 12;10(2):e24468. doi: 10.1016/j.heliyon.2024.e24468 (PMC10831622; doi:10.1016/j.heliyon.2024.e24468)
Supplement: Multimedia component 2 [file mmc2.docx]

Table S2 Nei's genetic distance of 9 cultivated varieties and 3 wild accessions of *S. ningpoensis.* The upper section presents the results of SRAP markers, the lower section displays the results of SSR markers.

| SRAP | TM | TT | LQ | FQ | DP | LZ | BYP | TB | LCP | DL | TD | GYX |
| --- | --- | --- | --- | --- | --- | --- | --- | --- | --- | --- | --- | --- |
| TM | 0.0000 |  |  |  |  |  |  |  |  |  |  |  |
| TT | 0.7130 | 0.0000 |  |  |  |  |  |  |  |  |  |  |
| LQ | 0.6609 | 0.6348 | 0.0000 |  |  |  |  |  |  |  |  |  |
| FQ | 0.4823 | 0.3629 | 0.3882 | 0.0000 |  |  |  |  |  |  |  |  |
| DP | 0.4683 | 0.3755 | 0.4011 | 0.0445 | 0.0000 |  |  |  |  |  |  |  |
| LZ | 0.5860 | 0.4545 | 0.3505 | 0.6783 | 0.6348 | 0.0000 |  |  |  |  |  |  |
| BYP | 0.5254 | 0.5705 | 0.4274 | 0.6435 | 0.6174 | 0.7043 | 0.0000 |  |  |  |  |  |
| TB | 0.5108 | 0.5553 | 0.4409 | 0.4823 | 0.4964 | 0.2906 | 0.2906 | 0.0000 |  |  |  |  |
| LCP | 0.5108 | 0.6178 | 0.4683 | 0.5652 | 0.5565 | 0.7130 | 0.7652 | 0.8087 | 0.0000 |  |  |  |
| DL | 0.4964 | 0.6018 | 0.4545 | 0.5565 | 0.5478 | 0.7217 | 0.2790 | 0.7826 | 0.0628 | 0.0000 |  |  |
| TD | 0.4683 | 0.5402 | 0.4274 | 0.6087 | 0.6000 | 0.3023 | 0.2563 | 0.8348 | 0.0628 | 0.0721 | 0.0000 |  |
| GYX | 0.4823 | 0.5553 | 0.4142 | 0.5826 | 0.5739 | 0.2676 | 0.3141 | 0.7913 | 0.0721 | 0.0815 | 0.9391 | 0.0000 |
| SSR | TM | TT | LQ | FQ | DP | LZ | BYP | TB | LCP | DL | TD | GYX |
| TM | 0.0000 |  |  |  |  |  |  |  |  |  |  |  |
| TT | 0.8750 | 0.0000 |  |  |  |  |  |  |  |  |  |  |
| LQ | 0.8125 | 0.8125 | 0.0000 |  |  |  |  |  |  |  |  |  |
| FQ | 0.3302 | 0.2469 | 0.5213 | 0.0000 |  |  |  |  |  |  |  |  |
| DP | 0.3302 | 0.2469 | 0.5213 | 0.0000 | 0.0000 |  |  |  |  |  |  |  |
| LZ | 0.4212 | 0.4212 | 0.3302 | 0.5625 | 0.5625 | 0.0000 |  |  |  |  |  |  |
| BYP | 0.3747 | 0.3747 | 0.5754 | 0.7188 | 0.7188 | 0.7188 | 0.0000 |  |  |  |  |  |
| TB | 0.3747 | 0.3747 | 0.3747 | 0.4212 | 0.4212 | 0.3302 | 0.2877 | 0.0000 |  |  |  |  |
| LCP | 0.3747 | 0.3747 | 0.3747 | 0.6562 | 0.6562 | 0.7812 | 0.7500 | 0.8750 | 0.0000 |  |  |  |
| DL | 0.4212 | 0.4212 | 0.4212 | 0.6875 | 0.6875 | 0.7500 | 0.2469 | 0.9062 | 0.0317 | 0.0000 |  |  |
| TD | 0.4212 | 0.4212 | 0.4212 | 0.6875 | 0.6875 | 0.2877 | 0.2469 | 0.9062 | 0.0317 | 0.0000 | 0.0000 |  |
| GYX | 0.4212 | 0.4212 | 0.4212 | 0.6875 | 0.6875 | 0.2877 | 0.2469 | 0.9062 | 0.0317 | 0.0000 | 1.0000 | 0.0000 |
