# Supplementary material for: The impact of non-environmental factors on the chemical variation of Radix Scrophulariae
Source: Heliyon. 2024 Jan 12;10(2):e24468. doi: 10.1016/j.heliyon.2024.e24468 (PMC10831622; doi:10.1016/j.heliyon.2024.e24468)
Supplement: Multimedia component 3 [file mmc3.docx]

Table S3 18 characteristic peak areas of different Radix *Scrophulariae* from nine cultivated varieties

| Retention time  Sample ID | 9.219 | 10.46 | 18.985 | 21.004 | 24.37 | 47.348 | 48.391 | 50.092 | 51.035 | 56.532 | 57.94 | 62.661 | 63.568 | 65.532 | 68 | 73.851 | 78 | 83.459 |
| --- | --- | --- | --- | --- | --- | --- | --- | --- | --- | --- | --- | --- | --- | --- | --- | --- | --- | --- |
| TD-upper | 63.85±6.47 | 178.69±48.68 | 151.2±7.1 | 57.4±1.11 | 47.29±2.04 | 62.32±11.72 | 246.46±2.08 | 100.03±1.75 | 639.02±13.55 | 60.44±5.42 | 45.69±3.23 | 26.35±0.57 | 465.83±17.74 | 344.59±14.67 | 14.84±8.43 | 40.08±2.1 | 80.22±18.59 | 27.61±16.98 |
| TD-middle | 86.21±7.76 | 86.21±7.76 | 86.21±7.76 | 86.21±7.76 | 86.21±7.76 | 86.21±7.76 | 86.21±7.76 | 86.21±7.76 | 86.21±7.76 | 86.21±7.76 | 86.21±7.76 | 86.21±7.76 | 86.21±7.76 | 86.21±7.76 | 86.21±7.76 | 86.21±7.76 | 86.21±7.76 | 86.21±7.76 |
| TD-lower | 73.75±19.03 | 126.11±5.39 | 220.84±2.7 | 138.96±8.33 | 126.62±3.11 | 60.35±0.42 | 166.98±2.08 | 28.42±5.41 | 688.13±0.82 | 46.19±9.68 | 39.68±2.23 | 39.39±0.55 | 797.18±13.05 | 385.82±1.2 | 69.53±0.65 | 110.85±1.78 | 58.55±0.37 | 35.19±7.01 |
| TD-basal buds | 58.73±26.33 | 254.84±4.12 | 177.5±2.4 | 57.61±0.48 | 107.85±1.14 | 48.48±0.02 | 247.02±31.06 | 77.34±9.31 | 885.37±17.22 | 57.86±15.48 | 65.08±3.76 | 29.37±12.66 | 382.55±3.2 | 276.71±1.75 | 24.27±0.62 | 22.06±10.23 | 27.03±12.85 | 33.99±3.44 |
| TD-whole | 75.44±6.79 | 129.93±11.69 | 126.24±11.36 | 108.37±9.75 | 101.48±9.13 | 47.54±4.28 | 255.60±23 | 35.87±3.23 | 66.88±6.02 | 513.61±46.23 | 170.94±15.38 | 633.29±57.00 | 311.09±28.00 | 45.96±4.14 | 0.00±0.00 | 72.59±6.53 | 23.24±2.09 | 121.06±10.9 |
| GYX-upper | 67.56±0.48 | 209.36±0.26 | 237.79±0.26 | 48.19±0.46 | 0.00±0.00 | 65.67±0.78 | 30.54±0.46 | 291.05±0.11 | 113.2±1.49 | 0.00±0.00 | 23.97±0.35 | 22.16±0.13 | 141.38±1.6 | 60.45±0.29 | 82.06±1.2 | 37.54±0.32 | 37.35±0.14 | 48.71±0.41 |
| GYX-middle | 53.00±4.77 | 53.00±4.77 | 53.00±4.77 | 53.00±4.77 | 53.00±4.77 | 53.00±4.77 | 53.00±4.77 | 53.00±4.77 | 53.00±4.77 | 53.00±4.77 | 53.00±4.77 | 53.00±4.77 | 53.00±4.77 | 53.00±4.77 | 53.00±4.77 | 53.00±4.77 | 53.00±4.77 | 53.00±4.77 |
| GYX-lower | 70.41±37.81 | 318.5±5.04 | 498.28±0.63 | 88.71±2.47 | 153.36±6.87 | 212.12±1.06 | 70.34±0.3 | 10.8±15.27 | 477.77±16.76 | 165.05±10.66 | 19.97±4.78 | 20.17±0.8 | 284.85±5.48 | 70.87±0.21 | 124.06±3.18 | 62.74±7.33 | 19.89±0.49 | 39.94±5.61 |
| GYX-basal buds | 132.68±1.08 | 505.27±41.53 | 229.03±9.25 | 53.03±2.48 | 14.19±20.07 | 233.29±7.89 | 129.81±5.29 | 10.69±15.12 | 528.31±15.09 | 180.76±12.13 | 26.81±6.8 | 22.77±1.13 | 88.2±1.33 | 50.93±4.03 | 105.58±1.54 | 37.76±1.38 | 50.87±1.23 | 30.62±4.70 |
| GYX-whole | 46.37±4.17 | 114.21±10.28 | 190.86±17.18 | 0.00±0.00 | 73.56±6.62 | 0.00±0.00 | 57.81±5.20 | 0.00±0.00 | 56.82±5.11 | 239.96±21.60 | 103.94±9.35 | 314.49±28.30 | 208.28±18.74 | 13.13±1.18 | 0.00±0.00 | 21.64±1.95 | 50.39±4.54 | 130.04±11.70 |
| BYP-upper | 71.41±9.42 | 106.48±18.37 | 109.63±1.67 | 107.28±10.01 | 0.00±0.00 | 35.25±2.00 | 217.46±2.88 | 114.53±2.06 | 362.89±9.88 | 127.69±1.04 | 25.5±0.48 | 0.00±0.00 | 300.39±3.54 | 264.71±0.23 | 58.92±7.91 | 32.13±0.61 | 51.8±4.94 | 23.95±1.23 |
| BYP-middle | 35.52±3.20 | 35.52±3.20 | 35.52±3.20 | 35.52±3.20 | 35.52±3.20 | 35.52±3.20 | 35.52±3.20 | 35.52±3.20 | 35.52±3.20 | 35.52±3.20 | 35.52±3.20 | 35.52±3.20 | 35.52±3.20 | 35.52±3.20 | 35.52±3.20 | 35.52±3.20 | 35.52±3.20 | 35.52±3.20 |
| BYP-lower | 69.06±0.58 | 105.21±1.65 | 206.26±0.44 | 180.23±0.63 | 50.93±0.08 | 472.78±2.69 | 33.23±0.52 | 129.85±7.62 | 32.68±5.26 | 730.23±16.41 | 213.26±19.7 | 41.89±0.52 | 641.59±0.02 | 296.86±4.25 | 38.52±0.6 | 32.56±0.77 | 44.99±0.46 | 24.84±0.94 |
| BYP-basal buds | 46.06±0.37 | 129.82±0.56 | 243.82±1.97 | 148.81±1.98 | 0.00±0.00 | 0.00±0.00 | 150.98±5.98 | 155.73±0.69 | 365.97±2.06 | 103.83±2 | 34.96±6.43 | 0.00±0.00 | 265.02±6.37 | 262.87±3.32 | 58.92±0.84 | 30.17±0.06 | 46.99±0.94 | 25.21±0.00 |
| BYP-whole | 31.08±2.80 | 108.79±9.79 | 100.22±9.02 | 68.35±6.15 | 13.21±1.19 | 0.00±0.00 | 222.84±20.06 | 0.00±0.00 | 107.47±9.67 | 350.00±31.50 | 109.39±9.85 | 229.76±20.68 | 55.24±4.97 | 0.00±0.00 | 0.00±0.00 | 0.00±0.00 | 31.40±2.83 | 47.71±4.29 |
| TB-upper | 49.49±0.28 | 175.08±0.76 | 187.29±0.33 | 82.71±17.26 | 0.00±0.00 | 63.94±0.07 | 171.88±0.46 | 74.07±0.35 | 261.04±337.43 | 331.31±224.76 | 111.76±102.54 | 27.32±15.6 | 390.19±1.74 | 216.9±0.23 | 23.4±1.82 | 47.77±27.88 | 49.67±25.65 | 53.26±9.35 |
| TB-middle | 35.25±3.17 | 35.25±3.17 | 35.25±3.17 | 35.25±3.17 | 35.25±3.17 | 35.25±3.17 | 35.25±3.17 | 35.25±3.17 | 35.25±3.17 | 35.25±3.17 | 35.25±3.17 | 35.25±3.17 | 35.25±3.17 | 35.25±3.17 | 35.25±3.17 | 35.25±3.17 | 35.25±3.17 | 35.25±3.17 |
| TB-lower | 37.25±0.01 | 84.61±0.35 | 203.74±0.84 | 174.3±1.62 | 37.48±0.27 | 63.22±0.52 | 176.93±2.27 | 63.42±0.94 | 718.77±19.02 | 205.98±16.53 | 45.26±3 | 50.39±0.48 | 1096.74±4.12 | 247.44±1.23 | 50.91±31.82 | 51.78±1.32 | 65.87±0.3 | 44.87±1.1 |
| TB-basal buds | 46.2±0.55 | 0.00±0.00 | 272.04±2.2 | 0.00±0.00 | 0.00±0.00 | 0.00±0.00 | 0.00±0.00 | 49.52±0.06 | 0.00±0.00 | 0.00±0.00 | 0.00±0.00 | 0.00±0.00 | 350.27±5.25 | 213.76±4.44 | 76.28±0.85 | 148.85±4.98 | 79.05±6.74 | 0.00±0.00 |
| TB-whole | 30.84±2.78 | 106.19±9.56 | 168.66±15.18 | 85.67±7.71 | 15.51±1.40 | 45.82±4.12 | 55.02±4.95 | 0.00±0.00 | 0.00±0.00 | 235.87±21.23 | 150.09±13.51 | 320.89±28.88 | 223.55±20.12 | 24.91±2.24 | 0.00±0.00 | 12.16±1.09 | 17.46±1.57 | 63.08±5.68 |
| LCP-upper | 128.28±1.61 | 246.21±0.13 | 236.23±0.15 | 269.4±0.16 | 31.24±0.03 | 81.31±0.17 | 574.5±0.5 | 83.51±0.13 | 32.65±0.71 | 79.91±0.7 | 193.19±1.09 | 29.52±0.53 | 1181.48±0.98 | 723.99±0.76 | 151.4±0.27 | 23.7±0.37 | 345.18±0.06 | 127.61±0.56 |
| LCP-middle | 40.43±3.64 | 40.43±3.64 | 40.43±3.64 | 40.43±3.64 | 40.43±3.64 | 40.43±3.64 | 40.43±3.64 | 40.43±3.64 | 40.43±3.64 | 40.43±3.64 | 40.43±3.64 | 40.43±3.64 | 40.43±3.64 | 40.43±3.64 | 40.43±3.64 | 40.43±3.64 | 40.43±3.64 | 40.43±3.64 |
| LCP-lower | 152.8±0.15 | 364.35±0.22 | 377.52±0.11 | 262.32±0.14 | 52.13±0.14 | 171.74±0.92 | 55.57±0.39 | 29.59±0.29 | 616.62±0.33 | 71.6±0.15 | 85.36±0.26 | 157.36±0.27 | 1139.5±0.37 | 963.37±0.09 | 25.57±0.04 | 59.98±0.59 | 59.09±0.18 | 37.57±2.22 |
| LCP-basal buds | 69.6±0.4 | 575.28±0.1 | 292.22±0.26 | 65.41±0.28 | 38.87±0.09 | 26.8±0.15 | 50.16±0.01 | 1329.41±0 | 98.83±0.58 | 46.65±0.67 | 360.66±0.63 | 27.66±0.29 | 1378.65±0.21 | 408.76±0.89 | 20.54±0.59 | 53.14±1.19 | 30.73±0.61 | 35.9±0.45 |
| LCP-whole | 35.38±3.18 | 95.26±8.57 | 196.62±17.70 | 84.62±7.62 | 13.43±1.21 | 0.00±0.00 | 102.56±9.23 | 0.00±0.00 | 237.1±21.34 | 414.75±37.33 | 133.31±12.00 | 248.38±22.35 | 37.16±3.34 | 79.68±7.17 | 0.00±0.00 | 6.66±0.60 | 32.25±2.90 | 46.81±4.21 |
| LZ-upper | 51.29±3.25 | 114.65±3.97 | 138.08±1.16 | 143.26±0.84 | 0.00±0.00 | 273.48±4.63 | 575.89±20.27 | 94.25±5.78 | 18.9±0.33 | 22.44±5.3 | 1002.33±1.63 | 53.11±37.48 | 930.99±18.83 | 550.9±7.83 | 63.26±3.22 | 52.14±10.1 | 30.55±10.12 | 0.00±0.00 |
| LZ-middle | 110.38±9.93 | 110.38±9.93 | 110.38±9.93 | 110.38±9.93 | 110.38±9.93 | 110.38±9.93 | 110.38±9.93 | 110.38±9.93 | 110.38±9.93 | 110.38±9.93 | 110.38±9.93 | 110.38±9.93 | 110.38±9.93 | 110.38±9.93 | 110.38±9.93 | 110.38±9.93 | 110.38±9.93 | 110.38±9.93 |
| LZ-lower | 235.31±0.14 | 222.46±0.34 | 94.77±0.23 | 41.75±0.08 | 57.23±0.23 | 110.85±0.22 | 625.18±0.17 | 37.48±0.32 | 100.38±0.18 | 33.13±0.11 | 1076.08±7.6 | 50.76±0.65 | 1093.16±3.86 | 118.19±1.59 | 22.18±0.09 | 51.01±0.84 | 21.74±0.33 | 0.00±0.00 |
| LZ-basal buds | 25.36±0.53 | 276.57±0.13 | 266.45±1.14 | 104.21±0.68 | 53.11±0.09 | 46.77±0.11 | 292.57±2.46 | 62.46±0.77 | 553.97±1.45 | 25.11±0.8 | 28.51±0.45 | 0.00±0.00 | 355.08±2.57 | 103.74±1.08 | 130.23±1.95 | 35.02±19.43 | 0.00±0.00 | 0.00±0.00 |
| LZ-whole | 96.58±8.69 | 185.75±16.72 | 185.69±16.71 | 103.63±9.33 | 69.09±6.22 | 24.04±2.16 | 193.63±17.43 | 89.72±8.07 | 46.03±4.14 | 693.94±62.45 | 47.83±4.3.00 | 561.10±50.50 | 53.42±4.81 | 21.07±1.90 | 117.78±10.60 | 42.45±3.82 | 22.21±2.00 | 0.00±0.00 |
| DL-upper | 27.48±1.66 | 449.51±2.17 | 385.35±0.85 | 176.6±4.31 | 159.49±0.92 | 0.00±0.00 | 493.87±9.72 | 85.39±1.89 | 0.00±0.00 | 0.00±0.00 | 0.00±0.00 | 0.00±0.00 | 588.73±6.84 | 311.94±3.74 | 165.45±3.2 | 33.49±0.95 | 51.23±9.41 | 30.56±0.49 |
| DL-middle | 57.60±5.18 | 57.60±5.18 | 57.60±5.18 | 57.60±5.18 | 57.60±5.18 | 57.60±5.18 | 57.60±5.18 | 57.60±5.18 | 57.60±5.18 | 57.60±5.18 | 57.60±5.18 | 57.60±5.18 | 57.60±5.18 | 57.60±5.18 | 57.60±5.18 | 57.60±5.18 | 57.60±5.18 | 57.60±5.18 |
| DL-lower | 119.88±5.29 | 657.17±36 | 452.92±9.78 | 262.67±1.87 | 250.58±5.71 | 88.44±1.75 | 467.74±22.74 | 28.02±2.19 | 69.7±3.75 | 79.96±0.95 | 0.00±0.00 | 0.00±0.00 | 1249.27±35.85 | 565.53±12.76 | 33.99±2.04 | 150.71±0.75 | 60.08±0.36 | 57.65±20.59 |
| DL-basal buds | 0.00±0.00 | 446.2±18.17 | 342.66±0.92 | 81.92±2.57 | 108.93±1.05 | 662±11.03 | 44.84±0.05 | 107.35±1.57 | 0.00±0.00 | 0.00±0.00 | 0.00±0.00 | 539.62±2.67 | 280.84±1.35 | 165.47±19.91 | 0.00±0.00 | 0.00±0.00 | 36.23±22.18 | 19.65±0.2 |
| DL-whole | 25.92±2.33 | 184.12±16.57 | 241.32±21.72 | 85.23±7.67 | 71.45±6.43 | 0.00±0.00 | 124.1±11.17 | 0.00±0.00 | 52.06±4.69 | 304.28±27.39 | 139.6±12.56 | 216.77±19.51 | 61.80±5.56 | 6.05±0.54 | 0.00±0.00 | 40.76±3.67 | 26.50±2.38 | 22.92±2.06 |
| FQ-upper | 253.73±23.97 | 377.21±21.52 | 165.25±10.13 | 143.66±1.19 | 133.87±6.29 | 37.57±1.61 | 56.15±2.8 | 302.7±0.62 | 28.75±1.57 | 27.75±2.08 | 155.46±3.42 | 36.15±1.27 | 1145.47±35.85 | 977.87±6.99 | 108.77±3.94 | 27.28±1.35 | 55.66±1.73 | 14.91±0.67 |
| FQ-middle | 237.13±21.34 | 375.04±33.75 | 215.51±19.40 | 153.22±13.79 | 212.78±19.15 | 95.11±8.56 | 245.87±22.13 | 23.97±2.16 | 9.46±0.85 | 568.40±51.16 | 713.25±64.19 | 1011.71±91.05 | 205.07±18.46 | 23.74±2.14 | 81.73±7.36 | 88.85±8.00 | 21.66±1.95 | 28.46±2.56 |
| FQ-lower | 215.59±0.56 | 463.38±7.02 | 337.24±0.76 | 128.45±1.37 | 261.26±0.02 | 56.46±0.37 | 242.28±0.2 | 49.48±0.09 | 79.94±1.35 | 798.69±0.41 | 61.5±0.94 | 58.67±0.02 | 862.52±0.63 | 71.5±0.56 | 95.4±0.5 | 49.9±0.01 | 99.46±0.17 | 35.92±0.03 |
| FQ-basal buds | 88.93±1.14 | 227.8±2.04 | 153.67±0.2 | 118.87±0.48 | 77.72±1.01 | 62.27±1.75 | 141.76±0.93 | 77.84±0.57 | 781.52±1.56 | 75.28±2.44 | 78.88±0.9 | 13.16±0.08 | 617.02±2.37 | 122.09±2.35 | 86.76±2.18 | 19.52±0.38 | 0.00±0.00 | 0.00±0.00 |
| FQ-whole | 207.49±18.67 | 314.57±28.31 | 265.18±23.87 | 134.07±12.07 | 186.18±16.76 | 83.22±7.49 | 227.91±20.51 | 20.97±1.89 | 8.28±0.75 | 529.09±47.62 | 624.10±56.17 | 860.79±77.47 | 159.57±14.36 | 20.77±1.87 | 71.51±6.44 | 77.75±7.00 | 18.95±1.71 | 24.91±2.24 |
| DP-upper | 272.4±0.04 | 368.58±1 | 161.06±1.1 | 146.61±0.52 | 139.37±0.21 | 39.66±0.3 | 54.22±0 | 274.39±5.61 | 30.48±0.08 | 35.97±0.62 | 140.35±0.23 | 42.49±0.04 | 1153.49±3.71 | 991.06±0.12 | 50.62±0.04 | 108.58±1.13 | 28.59±0.95 | 58.76±0.71 |
| DP-middle | 222.49±20.02 | 222.49±20.02 | 222.49±20.02 | 222.49±20.02 | 222.49±20.02 | 222.49±20.02 | 222.49±20.02 | 222.49±20.02 | 222.49±20.02 | 222.49±20.02 | 222.49±20.02 | 222.49±20.02 | 222.49±20.02 | 222.49±20.02 | 222.49±20.02 | 222.49±20.02 | 222.49±20.02 | 222.49±20.02 |
| DP-lower | 215.62±0.52 | 464.3±5.72 | 338.52±1.04 | 128.56±1.53 | 263.28±2.84 | 57.51±1.12 | 244.22±2.94 | 49.56±0.03 | 80.79±0.16 | 798.87±0.66 | 62.71±0.78 | 58.98±0.45 | 860.73±1.9 | 71.77±0.94 | 95.67±0.88 | 49.6±0.44 | 98.82±0.73 | 37.75±2.56 |
| DP-basal buds | 90.26±0.03 | 227.95±2.03 | 153.34±0.13 | 120.9±0.3 | 77.88±0.72 | 66.29±1.5 | 174.36±0.2 | 90.98±1.03 | 809.54±0.06 | 51.05±1.07 | 79.37±0.05 | 18.26±1.46 | 625.27±7 | 138.35±1.23 | 87.34±2.65 | 20.99±0.37 | 0.00±0.00 | 0.00±0.00 |
| DP-whole | 194.68±17.52 | 328.63±29.58 | 265.44±23.89 | 126.01±11.34 | 227.16±20.44 | 76.44±6.88 | 171.74±15.46 | 24.79±2.23 | 15.04±1.35 | 620.52±55.85 | 445.85±40.13 | 697.86±62.81 | 82.86±7.46 | 12.52±1.13 | 58.37±5.25 | 54.94±4.94 | 0.00±0.00 | 9.30±0.84 |
