# Supplementary material for: The impact of non-environmental factors on the chemical variation of Radix Scrophulariae
Source: Heliyon. 2024 Jan 12;10(2):e24468. doi: 10.1016/j.heliyon.2024.e24468 (PMC10831622; doi:10.1016/j.heliyon.2024.e24468)
Supplement: Multimedia component 4 [file mmc4.docx]

Table S4 The content of 6 components of different Radix *Scrophulariae* from nine cultivated varieties

|  | Varieties | Aucubin % | Harpagide % | Acteosde % | Angoroside C % | Harpagoside % | Cinnamic acid % |
| --- | --- | --- | --- | --- | --- | --- | --- |
| Whole underground part | FQ | 1.5020±0.3760ab | 0.7730±0.0830cdef | 0.1790±0.0530a | 0.5430±0.1060ab | 0.5400±0.0560a | 0.0330±0.0300abc |
|  | DP | 1.5830±0.3910a | 1.2060±0.1350ab | 0.1410±0.0330a | 0.3900±0.0890bcd | 0.4390±0.0670ab | 0.0170±0.0080bc |
|  | LZ | 0.8690±0.2640cd | 0.8460±0.0740bcdef | 0.1670±0.0470a | 0.5610±0.0220a | 0.3490±0.1140b | 0.0110±0.0070c |
|  | BYP | 0.4900±0.0590de | 0.4550±0.1650f | 0.1600±0.0830a | 0.2890±0.0530cde | 0.1450±0.0201cd | 0.0120±0.0070c |
|  | TB | 0.5890±0.1270de | 1.0570±0.0360abcd | 0.0470±0.0250b | 0.2150±0.0300def | 0.1550±0.0560cd | 0.0480±0.0020ab |
|  | LCP | 0.4520±0.1030e | 0.5690±0.1860ef | 0.1960±0.1620a | 0.3430±0.1410cde | 0.2130±0.0800c | 0.0210±0.0220bc |
|  | DL | 1.0280±0.1780bc | 1.3310±0.0860a | 0.0720±0.0080b | 0.2190±0.0360de | 0.1100±0.0360cd | 0.0100±0.0100c |
|  | TD | 0.6290±0.1150de | 0.5940±0.3180def | 0.1860±0.0520a | 0.4290±0.0710abc | 0.4050±0.0790ab | 0.0670±0.0120a |
|  | GYX | 0.5240±0.0840de | 0.8800±0.3000abcde | 0.0480±0.0130b | 0.1930±0.0370ef | 0.1980±0.0040c | 0.0430±0.0180ab |
| Dormant buds | FQ | 1.0376±0.0078g | 0.6985±0.0001g | 0.1125±0.0001h | 0.4414±0.0002e | 0.2043±0.0000e | 0.0716±0.0001a |
|  | DP | 1.0649±0.0013f | 0.7000±0.0007g | 0.1226±0.0018g | 0.6557±0.0044d | 0.3889±0.0010a | 0.0281±0.0006c |
|  | LZ | 1.3000±0.0004d | 1.2135±0.0050d | 0.2075±0.0018d | 0.7062±0.0003c | 0.2228±0.0016d | 0.0215±0.0003d |
|  | BYP | 0.9350±0.0071h | 0.7000±0.0141g | 0.8350±0.0071b | 0.9100±0.0141b | 0.1450±0.0071g | 0.0215±0.0021d |
|  | TB | 0.2171±0.0025i | 1.2388±0.0100c | 0.0351±0.0000i | 0.2848±0.0043f | 0.1341±0.0028h | 0.0158±0.0001e |
|  | LCP | 2.7041±0.0044a | 1.3405±0.0128b | 0.9428±0.0013a | 1.1241±0.0026a | 0.2572±0.0013b | 0.0112±0.0001f |
|  | DL | 2.1457±0.0190c | 1.5616±0.0042a | 0.4698±0.0078c | 0.4391±0.0022e | 0.1763±0.0008f | 0.0043±0.0000g |
|  | TD | 1.1971±0.0202e | 0.8079±0.0115f | 0.1799±0.0033e | 0.7194±0.0145c | 0.2399±0.0022c | 0.0573±0.0004b |
|  | GYX | 2.2256±0.0030b | 1.0136±0.0013e | 0.1687±0.0004f | 0.4329±0.0059e | 0.0553±0.0008i | 0.0102±0.0002f |
| Upper | FQ | 1.1578±0.0048c | 0.7395±0.0056e | 0.2465±0.0037d | 0.4419±0.0078e | 0.2498±0.0011e | 0.0710±0.0000b |
|  | DP | 1.3828±0.0018b | 0.7281±0.0118e | 0.2061±0.0125e | 0.4554±0.0053d | 0.5416±0.0008b | 0.0106±0.0001h |
|  | DL | 0.5254±0.0007g | 0.6287±0.0041f | 0.4186±0.0006a | 0.4504±0.0013de | 0.5840±0.0107a | 0.0722±0.0010a |
|  | LZ | 0.4393±0.0001h | 0.4990±0.0073g | 0.1541±0.0020g | 0.2916±0.0032g | 0.1739±0.0004g | 0.0549±0.0001d |
|  | BYP | 0.8230±0.0029e | 0.8531±0.0022d | 0.1219±0.0003h | 0.4025±0.0057f | 0.2448±0.0013e | 0.0450±0.0000e |
|  | TB | 1.1596±0.0065c | 1.0683±0.0065c | 0.4056±0.0016b | 1.0064±0.0076a | 0.4533±0.0007c | 0.0286±0.0001f |
|  | LCP | 2.1137±0.0105a | 1.7557±0.0041a | 0.3504±0.0070c | 0.4788±0.0055c | 0.1958±0.0023f | 0.0000±0.0000i |
|  | TD | 0.6873±0.0137f | 0.6211±0.0043f | 0.1750±0.0010f | 0.5192±0.0027b | 0.2886±0.0036d | 0.0674±0.0009c |
|  | GYX | 0.9844±0.0018d | 1.0887±0.0071b | 0.0463±0.0001i | 0.2371±0.0008h | 0.0901±0.0009h | 0.0126±0.0000g |
| Middle | FQ | 1.1070±0.0143b | 0.8824±0.0071e | 0.0804±0.0004f | 0.3232±0.0038g | 0.3540±0.0008e | 0.0710±0.0005c |
|  | DP | 1.0711±0.0052c | 0.8889±0.0035e | 0.0918±0.0003e | 0.5444±0.0050c | 0.6189±0.0035b | 0.0300±0.0003e |
|  | DL | 0.4909±0.0016g | 0.8607±0.0011g | 0.1891±0.0001c | 0.5148±0.0002d | 0.6497±0.0004a | 0.0805±0.0000a |
|  | LZ | 0.4507±0.0060h | 0.7012±0.0029h | 0.0945±0.0009d | 0.2824±0.0037h | 0.1884±0.0021g | 0.0751±0.0009b |
|  | BYP | 0.4921±0.0005g | 0.8723±0.0034f | 0.0692±0.0000g | 0.3969±0.0030f | 0.4679±0.0008d | 0.0581±0.0005d |
|  | TB | 1.0312±0.0013d | 1.2697±0.0019c | 0.3487±0.0001a | 0.9240±0.0002a | 0.6029±0.0004c | 0.0282±0.0000f |
|  | LCP | 1.9922±0.0018a | 2.0784±0.0033a | 0.3420±0.0016b | 0.5980±0.0001b | 0.3514±0.0031e | 0.0051±0.0000h |
|  | TD | 0.6605±0.0086f | 0.9806±0.0027d | 0.0670±0.0006h | 0.4043±0.0002e | 0.3317±0.0053f | 0.0704±0.0002c |
|  | GYX | 0.9650±0.0044e | 1.6383±0.0015b | 0.0664±0.0004h | 0.2532±0.0020h | 0.1270±0.0005h | 0.0162±0.0000g |
| Lower | FQ | 1.7792±0.0080c | 1.0479±0.0023e | 0.1414±0.0021g | 0.4421±0.0028a | 0.4389±0.0033e | 0.0801±0.0004b |
|  | DP | 1.8445±0.0025b | 1.5509±0.0171d | 0.1721±0.0004e | 0.6488±0.0007d | 0.6698±0.0020b | 0.0148±0.0001f |
|  | DL | 1.1050±0.0007f | 1.0129±0.0006f | 0.4427±0.0002a | 0.8739±0.0002c | 0.6869±0.0001a | 0.0887±0.0000a |
|  | LZ | 0.4930±0.0100h | 0.9398±0.0014g | 0.3354±0.0021d | 0.6022±0.0015e | 0.4027±0.0003f | 0.0616±0.0008c |
|  | BYP | 0.3978±0.0013i | 0.9280±0.0030g | 0.1255±0.0015h | 0.5946±0.0006f | 0.6882±0.0032a | 0.0513±0.0002e |
|  | TB | 1.7076±0.0005d | 1.7134±0.0003c | 0.4361±0.0001b | 0.9905±0.0004b | 0.6141±0.0018c | 0.0249±0.0000f |
|  | LCP | 2.2189±0.0161a | 2.0848±0.0123b | 0.3422±0.0011c | 1.0357±0.0004a | 0.3610±0.0009g | 0.0067±0.0000g |
|  | TD | 0.5745±0.0006g | 1.0051±0.0111f | 0.1184±0.0016i | 0.5591±0.0001g | 0.4998±0.0076d | 0.0799±0.0001b |
|  | GYX | 1.4788±0.0005e | 2.2683±0.0038a | 0.1504±0.0008f | 0.3796±0.0015i | 0.1787±0.0035h | 0.0147±0.0000f |

Footnote: Different lowercase letters represent significant differences
