# Supplementary material for: The impact of non-environmental factors on the chemical variation of Radix Scrophulariae
Source: Heliyon. 2024 Jan 12;10(2):e24468. doi: 10.1016/j.heliyon.2024.e24468 (PMC10831622; doi:10.1016/j.heliyon.2024.e24468)
Supplement: Multimedia component 5 [file mmc5.docx]

Table S5 The Euclidean distance of whole underground part of 9 cultivated varieties of *S. ningpoensis* based on 18 HPLC characteristic peak areas.

|  | FQ | DP | LZ | BYP | TB | LCP | DL | TD | GYX |
| --- | --- | --- | --- | --- | --- | --- | --- | --- | --- |
| FQ | 0 |  |  |  |  |  |  |  |  |
| DP | 2.491 | 0 |  |  |  |  |  |  |  |
| LZ | 5.840 | 5.312 | 0 |  |  |  |  |  |  |
| BYP | 8.035 | 7.681 | 5.791 | 0 |  |  |  |  |  |
| TB | 7.179 | 6.836 | 6.166 | 4.090 | 0 |  |  |  |  |
| LCP | 8.398 | 8.051 | 6.436 | 4.445 | 4.957 | 0 |  |  |  |
| DL | 6.561 | 6.036 | 5.317 | 3.530 | 3.470 | 4.512 | 0 |  |  |
| TD | 5.871 | 6.325 | 5.579 | 5.552 | 4.948 | 6.170 | 5.626 | 0 |  |
| GYX | 8.420 | 8.427 | 7.222 | 4.535 | 4.050 | 5.383 | 4.310 | 5.869 | 0 |
