# Supplementary material for: The impact of non-environmental factors on the chemical variation of Radix Scrophulariae
Source: Heliyon. 2024 Jan 12;10(2):e24468. doi: 10.1016/j.heliyon.2024.e24468 (PMC10831622; doi:10.1016/j.heliyon.2024.e24468)
Supplement: Multimedia component 6 [file mmc6.docx]

Table S6 The Euclidean distance of middle part of the roots of 9 cultivated varieties of *S. ningpoensis* based on 18 HPLC characteristic peak areas.

|  | FQ | DP | LZ | BYP | TB | LCP | DL | TD | GYX |
| --- | --- | --- | --- | --- | --- | --- | --- | --- | --- |
| FQ | 0 |  |  |  |  |  |  |  |  |
| DP | 2.874 | 0 |  |  |  |  |  |  |  |
| LZ | 6.415 | 6.127 | 0 |  |  |  |  |  |  |
| BYP | 7.467 | 7.506 | 5.431 | 0 |  |  |  |  |  |
| TB | 6.644 | 6.297 | 5.867 | 4.545 | 0 |  |  |  |  |
| LCP | 8.404 | 8.332 | 6.301 | 4.332 | 5.209 | 0 |  |  |  |
| DL | 6.439 | 5.543 | 5.292 | 4.641 | 4.131 | 5.675 | 0 |  |  |
| TD | 5.660 | 6.467 | 5.295 | 5.373 | 4.679 | 5.899 | 5.592 | 0 |  |
| GYX | 7.822 | 7.823 | 6.760 | 4.623 | 3.864 | 5.472 | 4.507 | 5.351 | 0 |
