# Supplementary material for: The impact of non-environmental factors on the chemical variation of Radix Scrophulariae
Source: Heliyon. 2024 Jan 12;10(2):e24468. doi: 10.1016/j.heliyon.2024.e24468 (PMC10831622; doi:10.1016/j.heliyon.2024.e24468)
Supplement: Multimedia component 7 [file mmc7.docx]

Table S7 The Euclidean distance of whole underground part of 9 cultivated varieties of *S. ningpoensis* based on 6 ingredients.

|  | FQ | DP | LZ | BYP | TB | LCP | DL | TD | GYX |
| --- | --- | --- | --- | --- | --- | --- | --- | --- | --- |
| FQ | 0 |  |  |  |  |  |  |  |  |
| DP | 1.408 | 0 |  |  |  |  |  |  |  |
| LZ | 2.818 | 2.271 | 0 |  |  |  |  |  |  |
| BYP | 4.775 | 4.616 | 3.057 | 0 |  |  |  |  |  |
| TB | 4.714 | 4.626 | 4.006 | 3.171 | 0 |  |  |  |  |
| LCP | 4.342 | 3.79 | 2.67 | 2.366 | 2.366 | 0 |  |  |  |
| DL | 3.79 | 3.308 | 3.072 | 2.921 | 2.516 | 1.481 | 0 |  |  |
| TD | 3.68 | 4.244 | 3.234 | 3.352 | 3.623 | 4.091 | 4.322 | 0 |  |
| GYX | 4.512 | 4.387 | 3.882 | 3.222 | 0.429 | 2.17 | 2.18 | 3.729 | 0 |
