# Supplementary material for: The impact of non-environmental factors on the chemical variation of Radix Scrophulariae
Source: Heliyon. 2024 Jan 12;10(2):e24468. doi: 10.1016/j.heliyon.2024.e24468 (PMC10831622; doi:10.1016/j.heliyon.2024.e24468)
Supplement: Multimedia component 8 [file mmc8.docx]

Table S8 The Euclidean distance of middle part of the roots of 9 cultivated varieties of *S. ningpoensis* based on 6 ingredients.

|  | FQ | DP | LZ | BYP | TB | LCP | DL | TD | GYX |
| --- | --- | --- | --- | --- | --- | --- | --- | --- | --- |
| FQ | 0 |  |  |  |  |  |  |  |  |
| DP | 2.105 | 0 |  |  |  |  |  |  |  |
| LZ | 2.633 | 2.288 | 0 |  |  |  |  |  |  |
| BYP | 3.928 | 4.453 | 3.218 | 0 |  |  |  |  |  |
| TB | 4.308 | 4.314 | 4.282 | 3.633 | 0 |  |  |  |  |
| LCP | 4.168 | 4.118 | 2.952 | 1.958 | 2.759 | 0 |  |  |  |
| DL | 4.097 | 3.024 | 3.596 | 3.657 | 2.975 | 3.053 | 0 |  |  |
| TD | 2.586 | 3.848 | 3.108 | 3.269 | 3.544 | 3.702 | 4.64 | 0 |  |
| GYX | 4.066 | 4.034 | 4.046 | 3.41 | 0.383 | 2.578 | 2.652 | 3.45 | 0 |
