# Supplementary material for: The impact of non-environmental factors on the chemical variation of Radix Scrophulariae
Source: Heliyon. 2024 Jan 12;10(2):e24468. doi: 10.1016/j.heliyon.2024.e24468 (PMC10831622; doi:10.1016/j.heliyon.2024.e24468)
Supplement: Multimedia component 9 [file mmc9.docx]

Table S9 The Euclidean distance matrix of each of the content of 6 ingredients based on the whole roots

| aucubin | FQ | DP | LZ | BYP | TB | LCP | DL | TD | GYX |
| --- | --- | --- | --- | --- | --- | --- | --- | --- | --- |
| FQ | 0 | 0.157 | 1.435 | 2.292 | 2.321 | 2.443 | 1.453 | 2.057 | 2.232 |
| DP | 0.157 | 0 | 1.592 | 2.449 | 2.478 | 2.599 | 1.61 | 2.213 | 2.388 |
| LZ | 1.435 | 1.592 | 0 | 0.857 | 0.886 | 1.008 | 0.018 | 0.622 | 0.797 |
| BYP | 2.292 | 2.449 | 0.857 | 0 | 0.029 | 0.151 | 0.839 | 0.236 | 0.06 |
| TB | 2.321 | 2.478 | 0.886 | 0.029 | 0 | 0.122 | 0.868 | 0.264 | 0.089 |
| LCP | 2.443 | 2.599 | 1.008 | 0.151 | 0.122 | 0 | 0.99 | 0.386 | 0.211 |
| DL | 1.453 | 1.61 | 0.018 | 0.839 | 0.868 | 0.99 | 0 | 0.604 | 0.779 |
| TD | 2.057 | 2.213 | 0.622 | 0.236 | 0.264 | 0.386 | 0.604 | 0 | 0.175 |
| GYX | 2.232 | 2.388 | 0.797 | 0.06 | 0.089 | 0.211 | 0.779 | 0.175 | 0 |
| harpagide | FQ | DP | LZ | BYP | TB | LCP | DL | TD | GYX |
| FQ | 0 | 0.005 | 1.381 | 2.865 | 1.676 | 1.191 | 0.414 | 2.413 | 1.291 |
| DP | 0.005 | 0 | 1.385 | 2.87 | 1.681 | 1.195 | 0.419 | 2.418 | 1.295 |
| LZ | 1.381 | 1.385 | 0 | 1.484 | 0.296 | 0.19 | 0.966 | 1.032 | 0.09 |
| BYP | 2.865 | 2.87 | 1.484 | 0 | 1.189 | 1.674 | 2.451 | 0.452 | 1.574 |
| TB | 1.676 | 1.681 | 0.296 | 1.189 | 0 | 0.486 | 1.262 | 0.737 | 0.385 |
| LCP | 1.191 | 1.195 | 0.19 | 1.674 | 0.486 | 0 | 0.776 | 1.222 | 0.1 |
| DL | 0.414 | 0.419 | 0.966 | 2.451 | 1.262 | 0.776 | 0 | 1.999 | 0.877 |
| TD | 2.413 | 2.418 | 1.032 | 0.452 | 0.737 | 1.222 | 1.999 | 0 | 1.122 |
| GYX | 1.291 | 1.295 | 0.09 | 1.574 | 0.385 | 0.1 | 0.877 | 1.122 | 0 |
| acteoside | FQ | DP | LZ | BYP | TB | LCP | DL | TD | GYX |
| FQ | 0 | 0.75 | 0.457 | 0.068 | 2.307 | 1.673 | 1.385 | 0.37 | 2.27 |
| DP | 0.75 | 0 | 0.292 | 0.682 | 1.557 | 0.923 | 0.636 | 1.119 | 1.52 |
| LZ | 0.457 | 0.292 | 0 | 0.39 | 1.85 | 1.215 | 0.928 | 0.827 | 1.812 |
| BYP | 0.068 | 0.682 | 0.39 | 0 | 2.239 | 1.605 | 1.318 | 0.437 | 2.202 |
| TB | 2.307 | 1.557 | 1.85 | 2.239 | 0 | 0.634 | 0.922 | 2.677 | 0.037 |
| LCP | 1.673 | 0.923 | 1.215 | 1.605 | 0.634 | 0 | 0.287 | 2.042 | 0.597 |
| DL | 1.385 | 0.636 | 0.928 | 1.318 | 0.922 | 0.287 | 0 | 1.755 | 0.885 |
| TD | 0.37 | 1.119 | 0.827 | 0.437 | 2.677 | 2.042 | 1.755 | 0 | 2.639 |
| GYX | 2.27 | 1.52 | 1.812 | 2.202 | 0.037 | 0.597 | 0.885 | 2.639 | 0 |
| angoroside C | FQ | DP | LZ | BYP | TB | LCP | DL | TD | GYX |
| FQ | 0 | 0.553 | 0.997 | 1.083 | 1.773 | 0.691 | 1.359 | 0.094 | 1.748 |
| DP | 0.553 | 0 | 0.444 | 1.635 | 2.325 | 1.244 | 1.912 | 0.646 | 2.301 |
| LZ | 0.997 | 0.444 | 0 | 2.079 | 2.769 | 1.688 | 2.356 | 1.09 | 2.745 |
| BYP | 1.083 | 1.635 | 2.079 | 0 | 0.69 | 0.391 | 0.276 | 0.989 | 0.665 |
| TB | 1.773 | 2.325 | 2.769 | 0.69 | 0 | 1.081 | 0.414 | 1.679 | 0.025 |
| LCP | 0.691 | 1.244 | 1.688 | 0.391 | 1.081 | 0 | 0.668 | 0.598 | 1.057 |
| DL | 1.359 | 1.912 | 2.356 | 0.276 | 0.414 | 0.668 | 0 | 1.266 | 0.389 |
| TD | 0.094 | 0.646 | 1.09 | 0.989 | 1.679 | 0.598 | 1.266 | 0 | 1.654 |
| GYX | 1.748 | 2.301 | 2.745 | 0.665 | 0.025 | 1.057 | 0.389 | 1.654 | 0 |
| harpagoside | FQ | DP | LZ | BYP | TB | LCP | DL | TD | GYX |
| FQ | 0 | 0.683 | 1.257 | 2.647 | 2.265 | 2.569 | 2.702 | 0.954 | 2.292 |
| DP | 0.683 | 0 | 0.574 | 1.964 | 1.581 | 1.886 | 2.018 | 0.271 | 1.608 |
| LZ | 1.257 | 0.574 | 0 | 1.39 | 1.008 | 1.312 | 1.444 | 0.303 | 1.035 |
| BYP | 2.647 | 1.964 | 1.39 | 0 | 0.382 | 0.078 | 0.055 | 1.693 | 0.355 |
| TB | 2.265 | 1.581 | 1.008 | 0.382 | 0 | 0.304 | 0.437 | 1.311 | 0.027 |
| LCP | 2.569 | 1.886 | 1.312 | 0.078 | 0.304 | 0 | 0.133 | 1.615 | 0.277 |
| DL | 2.702 | 2.018 | 1.444 | 0.055 | 0.437 | 0.133 | 0 | 1.747 | 0.41 |
| TD | 0.954 | 0.271 | 0.303 | 1.693 | 1.311 | 1.615 | 1.747 | 0 | 1.337 |
| GYX | 2.292 | 1.608 | 1.035 | 0.355 | 0.027 | 0.277 | 0.41 | 1.337 | 0 |
| cinnamic acid | FQ | DP | LZ | BYP | TB | LCP | DL | TD | GYX |
| FQ | 0 | 0.79 | 1.093 | 1.074 | 0.659 | 1.261 | 1.007 | 1.56 | 0.502 |
| DP | 0.79 | 0 | 0.303 | 0.284 | 1.449 | 0.471 | 0.217 | 2.35 | 1.292 |
| LZ | 1.093 | 0.303 | 0 | 0.019 | 1.752 | 0.167 | 0.086 | 2.654 | 1.595 |
| BYP | 1.074 | 0.284 | 0.019 | 0 | 1.733 | 0.186 | 0.068 | 2.635 | 1.576 |
| TB | 0.659 | 1.449 | 1.752 | 1.733 | 0 | 1.919 | 1.666 | 0.902 | 0.157 |
| LCP | 1.261 | 0.471 | 0.167 | 0.186 | 1.919 | 0 | 0.254 | 2.821 | 1.762 |
| DL | 1.007 | 0.217 | 0.086 | 0.068 | 1.666 | 0.254 | 0 | 2.567 | 1.508 |
| TD | 1.56 | 2.35 | 2.654 | 2.635 | 0.902 | 2.821 | 2.567 | 0 | 1.059 |
| GYX | 0.502 | 1.292 | 1.595 | 1.576 | 0.157 | 1.762 | 1.508 | 1.059 | 0 |
