# Supplementary material for: The impact of non-environmental factors on the chemical variation of Radix Scrophulariae
Source: Heliyon. 2024 Jan 12;10(2):e24468. doi: 10.1016/j.heliyon.2024.e24468 (PMC10831622; doi:10.1016/j.heliyon.2024.e24468)
Supplement: Multimedia component 10 [file mmc10.docx]

Table S10 The Euclidean distance matrix of each of the content of 6 ingredients based on the middle part of the roots

| aucubin | FQ | DP | LZ | BYP | TB | LCP | DL | TD | GYX |
| --- | --- | --- | --- | --- | --- | --- | --- | --- | --- |
| FQ | 0 | 0.138 | 1.281 | 2.2 | 2.404 | 2.343 | 0.915 | 2.03 | 2.125 |
| DP | 0.138 | 0 | 1.42 | 2.338 | 2.542 | 2.481 | 1.053 | 2.168 | 2.263 |
| LZ | 1.281 | 1.42 | 0 | 0.919 | 1.123 | 1.062 | 0.366 | 0.749 | 0.843 |
| BYP | 2.2 | 2.338 | 0.919 | 0 | 0.204 | 0.143 | 1.285 | 0.17 | 0.075 |
| TB | 2.404 | 2.542 | 1.123 | 0.204 | 0 | 0.061 | 1.489 | 0.374 | 0.279 |
| LCP | 2.343 | 2.481 | 1.062 | 0.143 | 0.061 | 0 | 1.428 | 0.313 | 0.218 |
| DL | 0.915 | 1.053 | 0.366 | 1.285 | 1.489 | 1.428 | 0 | 1.115 | 1.21 |
| TD | 2.03 | 2.168 | 0.749 | 0.17 | 0.374 | 0.313 | 1.115 | 0 | 0.095 |
| GYX | 2.125 | 2.263 | 0.843 | 0.075 | 0.279 | 0.218 | 1.21 | 0.095 | 0 |
| harpagide | FQ | DP | LZ | BYP | TB | LCP | DL | TD | GYX |
| FQ | 0 | 1.54 | 0.212 | 1.162 | 0.334 | 0.788 | 1.773 | 0.706 | 0.362 |
| DP | 1.54 | 0 | 1.328 | 2.702 | 1.207 | 2.329 | 0.232 | 2.247 | 1.179 |
| LZ | 0.212 | 1.328 | 0 | 1.374 | 0.121 | 1.001 | 1.56 | 0.919 | 0.149 |
| BYP | 1.162 | 2.702 | 1.374 | 0 | 1.495 | 0.373 | 2.934 | 0.455 | 1.523 |
| TB | 0.334 | 1.207 | 0.121 | 1.495 | 0 | 1.122 | 1.439 | 1.04 | 0.028 |
| LCP | 0.788 | 2.329 | 1.001 | 0.373 | 1.122 | 0 | 2.561 | 0.082 | 1.15 |
| DL | 1.773 | 0.232 | 1.56 | 2.934 | 1.439 | 2.561 | 0 | 2.479 | 1.411 |
| TD | 0.706 | 2.247 | 0.919 | 0.455 | 1.04 | 0.082 | 2.479 | 0 | 1.068 |
| GYX | 0.362 | 1.179 | 0.149 | 1.523 | 0.028 | 1.15 | 1.411 | 1.068 | 0 |
| acteoside | FQ | DP | LZ | BYP | TB | LCP | DL | TD | GYX |
| FQ | 0 | 0.808 | 0.381 | 0.258 | 2.325 | 1.589 | 1.469 | 0.188 | 2.15 |
| DP | 0.808 | 0 | 0.427 | 1.066 | 1.517 | 0.781 | 0.661 | 0.996 | 1.342 |
| LZ | 0.381 | 0.427 | 0 | 0.639 | 1.944 | 1.208 | 1.088 | 0.569 | 1.769 |
| BYP | 0.258 | 1.066 | 0.639 | 0 | 2.583 | 1.847 | 1.727 | 0.07 | 2.408 |
| TB | 2.325 | 1.517 | 1.944 | 2.583 | 0 | 0.736 | 0.856 | 2.513 | 0.175 |
| LCP | 1.589 | 0.781 | 1.208 | 1.847 | 0.736 | 0 | 0.12 | 1.777 | 0.561 |
| DL | 1.469 | 0.661 | 1.088 | 1.727 | 0.856 | 0.12 | 0 | 1.657 | 0.681 |
| TD | 0.188 | 0.996 | 0.569 | 0.07 | 2.513 | 1.777 | 1.657 | 0 | 2.337 |
| GYX | 2.15 | 1.342 | 1.769 | 2.408 | 0.175 | 0.561 | 0.681 | 2.337 | 0 |
| angoroside C | FQ | DP | LZ | BYP | TB | LCP | DL | TD | GYX |
| FQ | 0 | 0.515 | 1.368 | 0.936 | 1.571 | 0.472 | 1.2 | 0.162 | 1.521 |
| DP | 0.515 | 0 | 0.853 | 1.452 | 2.086 | 0.987 | 1.715 | 0.353 | 2.037 |
| LZ | 1.368 | 0.853 | 0 | 2.304 | 2.939 | 1.84 | 2.568 | 1.206 | 2.889 |
| BYP | 0.936 | 1.452 | 2.304 | 0 | 0.634 | 0.465 | 0.263 | 1.099 | 0.585 |
| TB | 1.571 | 2.086 | 2.939 | 0.634 | 0 | 1.099 | 0.371 | 1.733 | 0.049 |
| LCP | 0.472 | 0.987 | 1.84 | 0.465 | 1.099 | 0 | 0.728 | 0.634 | 1.05 |
| DL | 1.2 | 1.715 | 2.568 | 0.263 | 0.371 | 0.728 | 0 | 1.362 | 0.322 |
| TD | 0.162 | 0.353 | 1.206 | 1.099 | 1.733 | 0.634 | 1.362 | 0 | 1.684 |
| GYX | 1.521 | 2.037 | 2.889 | 0.585 | 0.049 | 1.05 | 0.322 | 1.684 | 0 |
| harpagoside | FQ | DP | LZ | BYP | TB | LCP | DL | TD | GYX |
| FQ | 0 | 0.684 | 1.198 | 2.578 | 2.101 | 2.51 | 2.798 | 0.792 | 2.18 |
| DP | 0.684 | 0 | 0.514 | 1.894 | 1.417 | 1.826 | 2.114 | 0.108 | 1.496 |
| LZ | 1.198 | 0.514 | 0 | 1.38 | 0.903 | 1.312 | 1.6 | 0.406 | 0.982 |
| BYP | 2.578 | 1.894 | 1.38 | 0 | 0.477 | 0.068 | 0.22 | 1.786 | 0.398 |
| TB | 2.101 | 1.417 | 0.903 | 0.477 | 0 | 0.409 | 0.697 | 1.309 | 0.079 |
| LCP | 2.51 | 1.826 | 1.312 | 0.068 | 0.409 | 0 | 0.288 | 1.718 | 0.33 |
| DL | 2.798 | 2.114 | 1.6 | 0.22 | 0.697 | 0.288 | 0 | 2.006 | 0.618 |
| TD | 0.792 | 0.108 | 0.406 | 1.786 | 1.309 | 1.718 | 2.006 | 0 | 1.388 |
| GYX | 2.18 | 1.496 | 0.982 | 0.398 | 0.079 | 0.33 | 0.618 | 1.388 | 0 |
| cinnamic acid | FQ | DP | LZ | BYP | TB | LCP | DL | TD | GYX |
| FQ | 0 | 0.808 | 1.339 | 1.286 | 0.617 | 1.489 | 1.174 | 1.174 | 0.448 |
| DP | 0.808 | 0 | 0.531 | 0.477 | 1.426 | 0.68 | 0.366 | 1.982 | 1.256 |
| LZ | 1.339 | 0.531 | 0 | 0.054 | 1.956 | 0.149 | 0.165 | 2.513 | 1.787 |
| BYP | 1.286 | 0.477 | 0.054 | 0 | 1.903 | 0.203 | 0.111 | 2.459 | 1.733 |
| TB | 0.617 | 1.426 | 1.956 | 1.903 | 0 | 2.106 | 1.792 | 0.557 | 0.169 |
| LCP | 1.489 | 0.68 | 0.149 | 0.203 | 2.106 | 0 | 0.314 | 2.662 | 1.936 |
| DL | 1.174 | 0.366 | 0.165 | 0.111 | 1.792 | 0.314 | 0 | 2.348 | 1.622 |
| TD | 1.174 | 1.982 | 2.513 | 2.459 | 0.557 | 2.662 | 2.348 | 0 | 0.726 |
| GYX | 0.448 | 1.256 | 1.787 | 1.733 | 0.169 | 1.936 | 1.622 | 0.726 | 0 |
