# Supplementary material for: The impact of non-environmental factors on the chemical variation of Radix Scrophulariae
Source: Heliyon. 2024 Jan 12;10(2):e24468. doi: 10.1016/j.heliyon.2024.e24468 (PMC10831622; doi:10.1016/j.heliyon.2024.e24468)
Supplement: Multimedia component 11 [file mmc11.docx]

Table S11 18 characteristic peak areas of different Radix *Scrophulariae* from same variety of DP

| Retention time  Sample ID | 9.219 | 10.46 | 18.985 | 21.004 | 24.37 | 47.348 | 48.391 | 50.092 | 51.035 | 56.532 | 57.94 | 62.661 | 63.568 | 65.532 | 68 | 73.851 | 78 | 83.459 |
| --- | --- | --- | --- | --- | --- | --- | --- | --- | --- | --- | --- | --- | --- | --- | --- | --- | --- | --- |
| DP-1 | 149.09±2.5 | 158.74±1.69 | 189.16±0.36 | 227.73±1.47 | 122.47±0.09 | 63.17±1.21 | 247.8±0.71 | 46.72±2.69 | 86.54±0.34 | 28.31±0.18 | 616.37±6.95 | 187.31±0.56 | 894.55±5.34 | 467.39±4.07 | 71.41±0.94 | 78.18±1.34 | 133.51±2.26 | 150.48±3.83 |
| DP-2 | 117.46±2.31 | 114.76±3.08 | 165.34±0.96 | 132.63±1.01 | 89.97±0.26 | 70.78±0.68 | 117.47±1.11 | 29.05±0.51 | 322.69±0.13 | 131.62±1.76 | 18.4±3.46 | 26.74±10 | 424.82±4.11 | 341.66±6.53 | 38±0.17 | 63.75±0.46 | 45.53±5.51 | 108.66±26.96 |
| DP-3 | 73.59±0.06 | 226.01±2.28 | 143.66±4.02 | 144.93±2.54 | 128.47±1.69 | 45.37±0.01 | 423.2±3.07 | 84.03±0.02 | 98.41±1.05 | 430.15±1.04 | 628.99±0.72 | 161.31±11.25 | 1138.85±9.08 | 268.86±5.67 | 65.7±15.89 | 97.15±1.35 | 149.13±3.1 | 126.47±18.98 |
| DP-4 | 106.55±3.2 | 159.09±0.76 | 207.31±0.55 | 151.76±0.14 | 92.78±2.21 | 50.05±0.02 | 390.06±2.39 | 43.02±1.1 | 98.68±3.79 | 29.51±5.99 | 723.15±8.23 | 200.95±5.85 | 852.55±3.86 | 350.75±0.46 | 62.11±7 | 72.71±4.83 | 71.33±6.89 | 193.1±2.55 |
| DP-5 | 90.87±0.18 | 92.46±1.58 | 147.57±3.02 | 129.77±0.34 | 114.21±1.04 | 58.32±0.3 | 158.42±0.24 | 35.59±1.31 | 41.66±2.17 | 27.24±1.28 | 529.13±14.95 | 216.09±13.05 | 793.62±1.24 | 303.27±1.09 | 49.8±2.82 | 60.75±14.29 | 120.2±8.3 | 123.76±5.6 |
| DP-6 | 134.69±2.03 | 138.42±2.23 | 142.62±0.65 | 149.8±5.01 | 97.27±1.34 | 62.06±0.49 | 462.69±4.57 | 42.36±1.18 | 167.47±1.35 | 35.82±2.14 | 839.47±16.87 | 214.95±6.53 | 1029.9±16.14 | 400.2±0.67 | 88.46±0.93 | 101.42±35.06 | 206.54±41.45 | 125.44±9.01 |
| DP-7 | 113.51±0.75 | 90.32±2.9 | 145.56±1.47 | 78.77±1.77 | 44±0.03 | 72.99±0.31 | 228.55±2.52 | 46.08±13.12 | 47.81±278.19 | 26.78±202.9 | 492.54±95.33 | 150.72±16.61 | 492.6±10.34 | 594.82±1.94 | 33.58±16.38 | 84.25±21.26 | 31.26±4.58 | 283.45±3.64 |
| DP-8 | 58.8±0.23 | 199.39±2.73 | 279.43±1.07 | 93.93±0.67 | 160.66±0.78 | 59.39±0.48 | 214.99±0.38 | 43.66±1.2 | 66.52±0.15 | 30.86±1.05 | 565.54±5.08 | 216.73±5.38 | 897.04±1.28 | 338.85±2.51 | 60.69±0.35 | 88.84±2.03 | 185.89±4.25 | 238.99±5.88 |
| DP-9 | 70.53±9.55 | 85.02±20.25 | 184.09±2.89 | 107.3±12.19 | 80.08±5.18 | 42.74±0.71 | 180.46±3.42 | 65.59±0.7 | 525.64±0.41 | 191.75±8.42 | 35.68±0.14 | 27.47±13.7 | 616.82±7.93 | 230.26±2.75 | 75.07±3.21 | 90.61±1.91 | 79.07±0.02 | 111.04±1.66 |
| DP-10 | 74.02±1.93 | 91.61±2.57 | 151.27±0.8 | 87.38±0.84 | 70.67±0.22 | 70.54±0.56 | 200.58±0.92 | 51.86±0.42 | 65.79±0.11 | 26.25±1.47 | 587.51±2.88 | 176.73±8.33 | 565.28±3.43 | 363.89±5.44 | 97.17±0.14 | 41.32±0.38 | 101.73±4.59 | 748.7±22.47 |
| DP-11 | 102.72±0.05 | 148.51±1.9 | 150.57±3.35 | 113.27±2.12 | 107.15±1.41 | 65.97±0.01 | 260.46±2.56 | 46.19±0.02 | 66.33±0.87 | 25.52±0.87 | 630.35±0.6 | 182.37±9.38 | 677.15±7.56 | 438.1±4.73 | 51.65±13.25 | 75.36±1.12 | 67.64±2.58 | 219.92±15.82 |
| DP-12 | 73.43±2.66 | 200.67±0.63 | 242.7±0.46 | 74.24±0.12 | 93.15±1.84 | 50.32±0.01 | 153.09±1.99 | 35.93±0.91 | 244.23±3.16 | 324.56±4.99 | 92.55±6.85 | 29.62±4.87 | 712.73±3.21 | 317.75±0.38 | 34.66±5.83 | 61.82±4.03 | 163.31±5.74 | 117.14±2.12 |
| DP-13 | 186.71±0.15 | 254.36±1.32 | 196.02±2.52 | 167.61±0.29 | 154.89±0.87 | 55.36±0.25 | 125.38±0.2 | 34.22±1.09 | 50.09±1.81 | 674.96±1.07 | 60.68±12.46 | 57.35±10.87 | 1071.46±1.03 | 145.9±0.91 | 28.83±2.35 | 99.18±11.91 | 66.04±6.92 | 148.93±4.67 |
| DP-14 | 215.98±1.69 | 468.34±1.86 | 337.78±0.55 | 127.48±4.18 | 261.27±1.12 | 56.72±0.4 | 242.14±3.81 | 49.54±0.98 | 80.9±1.12 | 798.4±1.78 | 62.16±14.06 | 58.66±5.44 | 862.08±13.45 | 71.11±0.56 | 95.05±0.78 | 49.91±29.22 | 99.34±34.54 | 35.94±7.51 |
| DP-15 | 90.24±0.63 | 226.51±2.42 | 153.43±1.23 | 121.11±1.48 | 77.37±0.02 | 65.23±0.26 | 174.5±2.1 | 91.71±10.93 | 809.5±231.82 | 51.81±169.08 | 79.34±79.44 | 17.23±13.85 | 620.32±8.61 | 137.48±1.62 | 85.47±13.65 | 20.73±17.72 | 0.00±0.00 | 0.00±0.00 |
| DP-16 | 215.98±0.19 | 468.34±2.27 | 337.78±0.89 | 127.48±0.56 | 261.27±0.65 | 56.72±0.4 | 242.14±0.31 | 49.54±1 | 80.9±0.13 | 798.4±0.87 | 62.16±4.24 | 58.66±4.48 | 862.08±1.06 | 71.11±2.09 | 95.05±0.29 | 49.91±1.69 | 99.34±3.54 | 35.94±4.9 |
| DP-17 | 89.74±7.96 | 226.35±16.88 | 153.81±2.41 | 119.21±10.16 | 77±4.31 | 61.04±0.59 | 141.1±2.85 | 77.43±0.58 | 782.62±0.34 | 77.01±7.02 | 78.24±0.12 | 13.1±11.42 | 618.7±6.61 | 123.75±2.29 | 88.3±2.68 | 19.79±1.59 | 0.00±0.00 | 0.00±0.00 |
| DP-18 | 270.68±3.12 | 362±34.68 | 158.08±12.01 | 144.5±12.71 | 138.32±10.58 | 38.71±4.2 | 54.16±35.18 | 303.14±65.63 | 29.86±73.94 | 26.28±459.99 | 153.05±430.83 | 37.05±12.54 | 1170.82±10.03 | 982.82±28.07 | 111.55±3.04 | 28.23±9.3 | 56.88±13.51 | 15.38±0.75 |
| DP-19 | 219.89±2.05 | 294.37±0.68 | 194.19±1.47 | 172.79±2.49 | 153.23±0.55 | 54.67±0.09 | 129.7±7.32 | 33.23±0.48 | 51.02±2.09 | 645.03±0.74 | 89.55±13.82 | 55.47±6.23 | 1065.12±11.54 | 143.73±3.13 | 29.39±1.24 | 104.28±25.68 | 65.85±28.23 | 149.52±9.22 |
| DP-20 | 272.37±2.19 | 367.87±5.24 | 161.84±1.46 | 146.98±2.24 | 139.22±0 | 39.87±0.22 | 54.22±0.05 | 278.36±0.21 | 30.54±0.26 | 35.53±0.74 | 140.19±4.6 | 42.46±0.15 | 1156.12±6.37 | 991.15±0.32 | 50.65±0.26 | 107.78±0.73 | 27.92±1.4 | 58.25±0.28 |
