# Supplementary material for: The impact of non-environmental factors on the chemical variation of Radix Scrophulariae
Source: Heliyon. 2024 Jan 12;10(2):e24468. doi: 10.1016/j.heliyon.2024.e24468 (PMC10831622; doi:10.1016/j.heliyon.2024.e24468)
Supplement: Multimedia component 12 [file mmc12.docx]

Table S12 18 characteristic peak areas of Radix *Scrophulariae* from the middle part of the roots of S. *ningpoensis* of DP variety

| Sample  Retention time | DP-1 | DP-2 | DP-3 | DP-4 | DP-5 | DP-6 | DP-7 | DP-8 | DP-9 | DP-10 | DP-11 | DP-12 |
| --- | --- | --- | --- | --- | --- | --- | --- | --- | --- | --- | --- | --- |
| 9.219 | 103.19±2.12 | 53.46±0.05 | 64.12±2.93 | 67.29±0.16 | 93.38±1.86 | 66.75±0.69 | 135.54±0.21 | 106.78±8.75 | 66.9±3.43 | 96.86±2.26 | 82.61±2.41 | 122.45±2.75 |
| 10.46 | 82.11±2.82 | 181.26±2.09 | 77.29±0.69 | 83.28±1.45 | 135.01±2.04 | 182.43±2.66 | 144.31±2.5 | 104.33±18.57 | 205.46±38.15 | 144.63±0.75 | 84.05±5.77 | 125.84±1.86 |
| 18.985 | 132.33±0.88 | 254.02±3.68 | 167.35±0.5 | 137.52±2.77 | 136.89±0.6 | 220.64±1.35 | 171.97±0.98 | 150.31±2.65 | 130.6±13.21 | 188.46±1.62 | 134.16±1.61 | 129.65±0.4 |
| 21.004 | 71.61±0.92 | 85.39±2.33 | 97.55±0.13 | 79.44±0.31 | 102.97±4.59 | 67.49±1.63 | 207.03±0.62 | 120.57±11.17 | 131.75±13.98 | 137.96±2.74 | 117.97±2.46 | 136.18±1.62 |
| 24.37 | 40±0.24 | 146.05±1.55 | 72.8±2.02 | 64.25±0.96 | 97.41±1.23 | 84.68±0.02 | 111.33±0.72 | 81.79±4.74 | 116.79±11.64 | 84.34±0.61 | 103.83±0.01 | 88.42±0.1 |
| 47.348 | 66.35±0.62 | 53.99±0.01 | 38.86±0.01 | 64.12±0.28 | 59.98±0.44 | 45.75±0.28 | 57.43±0.44 | 64.35±0.65 | 41.25±4.62 | 45.5±0.1 | 53.02±0.25 | 56.41±1.33 |
| 48.391 | 207.77±1.02 | 195.45±2.82 | 164.05±2.19 | 182.34±0.22 | 236.79±4.19 | 139.17±2.31 | 225.27±0.35 | 106.79±3.13 | 384.73±38.7 | 354.6±8.05 | 144.01±0.05 | 420.63±0.78 |
| 50.092 | 41.89±0.46 | 39.69±0.02 | 59.63±1.01 | 47.14±1.2 | 41.99±1.08 | 32.66±12.02 | 42.48±1.1 | 26.41±0.64 | 76.39±72.19 | 39.11±0.53 | 32.36±0.24 | 38.51±2.96 |
| 51.035 | 43.47±0.12 | 60.47±0.96 | 477.86±3.47 | 59.81±1.99 | 60.3±1.24 | 222.02±255.01 | 78.68±0.14 | 293.36±0.37 | 89.46±81.34 | 89.71±2.3 | 37.87±0.28 | 152.24±0.38 |
| 56.532 | 24.35±1.61 | 28.06±0.96 | 174.31±5.49 | 23.87±1.18 | 23.2±1.96 | 295.06±185.99 | 25.74±0.96 | 119.65±7.72 | 391.04±505.99 | 26.83±0.82 | 24.76±0.82 | 32.56±0.19 |
| 57.94 | 447.77±3.17 | 514.13±0.66 | 32.44±7.54 | 534.1±13.7 | 573.05±15.46 | 84.13±87.39 | 560.34±4.66 | 16.73±0.13 | 571.81±473.92 | 657.41±15.21 | 481.03±5.06 | 763.15±7.65 |
| 62.661 | 137.02±9.17 | 197.03±10.31 | 24.97±5.36 | 160.66±11.96 | 165.79±5.99 | 26.92±15.23 | 170.28±4.93 | 24.31±12.56 | 146.64±137.97 | 182.68±6.85 | 196.45±0.17 | 195.41±0.62 |
| 63.568 | 447.81±3.77 | 815.49±8.32 | 560.75±3.53 | 513.89±1.14 | 615.59±14.8 | 647.93±9.47 | 813.22±1.17 | 386.2±7.27 | 1035.32±110.32 | 775.04±12.69 | 721.48±7 | 936.27±5.88 |
| 65.532 | 540.74±5.98 | 308.05±5.2 | 209.33±0.42 | 330.81±1 | 398.27±0.61 | 288.86±1.78 | 424.9±2.3 | 310.6±2.52 | 244.42±30.88 | 318.87±3.45 | 275.7±0.36 | 363.82±4.48 |
| 68 | 30.53±0.16 | 55.17±14.57 | 68.25±6.42 | 88.34±2.58 | 46.96±0.85 | 31.51±15.02 | 64.92±0.32 | 34.54±2.94 | 59.72±3.35 | 56.46±1.37 | 45.28±0.29 | 80.42±1.03 |
| 73.851 | 76.59±0.42 | 80.76±1.24 | 82.38±44.31 | 37.57±13.1 | 68.51±32.14 | 56.2±19.49 | 71.07±1.86 | 57.95±1.75 | 88.32±10.23 | 66.1±28.25 | 55.22±0.8 | 92.2±1.48 |
| 78 | 28.42±5.05 | 168.99±2.84 | 71.88±63.19 | 92.48±76.09 | 61.49±37.99 | 148.46±4.2 | 121.37±3.9 | 41.39±0.02 | 135.58±14.86 | 64.85±31.05 | 109.28±1.55 | 187.76±2.49 |
| 83.459 | 257.68±24.72 | 217.27±174 | 100.94±2.34 | 680.64±513.2 | 199.92±8.26 | 106.49±3.34 | 136.8±5.39 | 98.78±1.52 | 114.98±0.82 | 175.55±10.14 | 112.51±0.31 | 114.04±4.21 |
| length/cm | 26 | 23 | 18 | 17 | 16 | 15 | 14 | 14 | 13 | 13 | 12 | 12 |
| diameter/cm | 5 | 4.8 | 4.3 | 4.5 | 4.6 | 3.8 | 4 | 4 | 4 | 4 | 3.8 | 3.8 |
| weight/g | 44.153 | 33.802 | 28.456 | 26.897 | 26.029 | 21.468 | 19.829 | 19.747 | 16.428 | 16.006 | 12.1 | 10.714 |
