# Supplementary figures and images for: The impact of non-environmental factors on the chemical variation of Radix Scrophulariae
Source: Heliyon. 2024 Jan 12;10(2):e24468. doi: 10.1016/j.heliyon.2024.e24468 (PMC10831622; doi:10.1016/j.heliyon.2024.e24468)

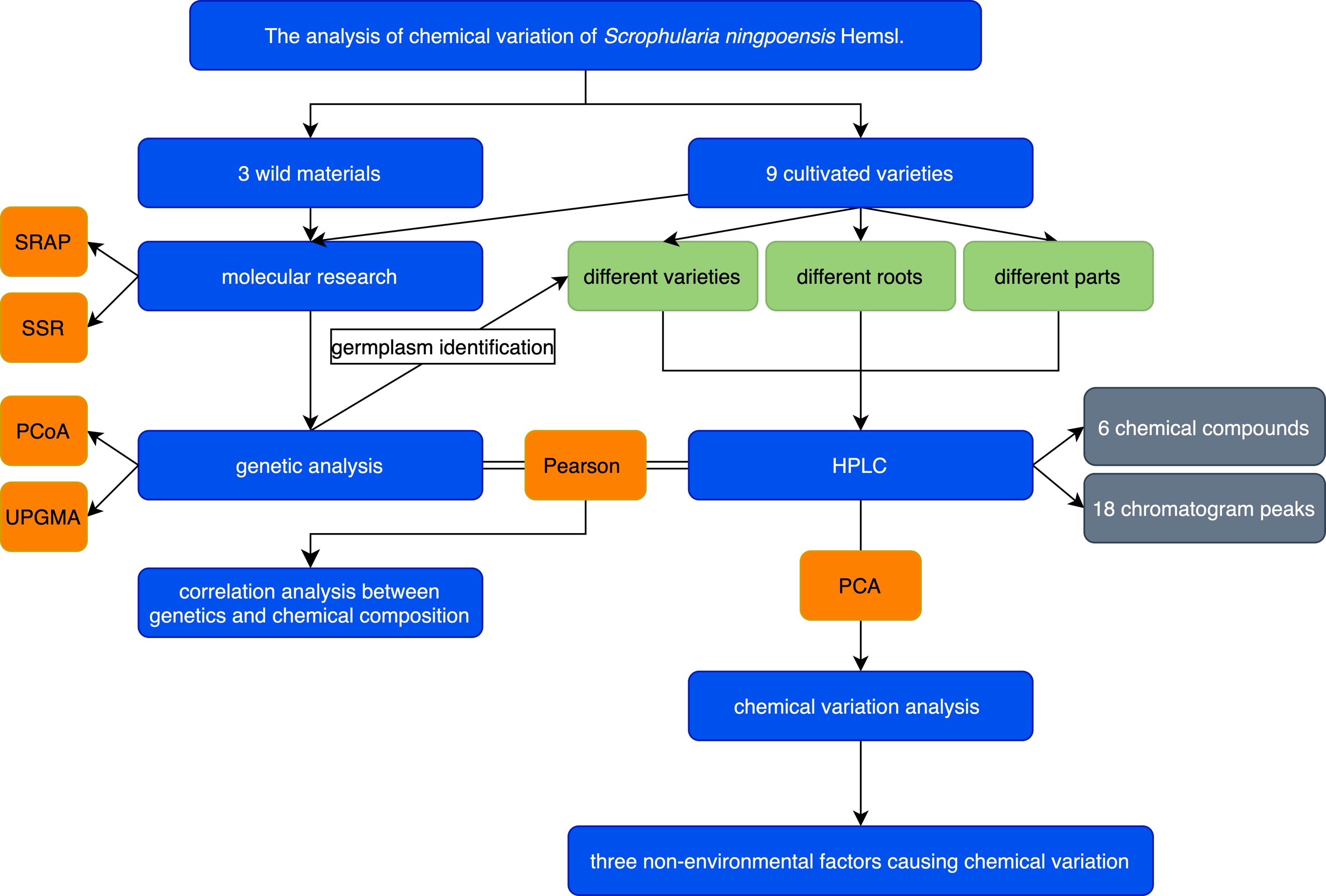


Fig. S1. The flow chart of the experimental design of this study

Supplement: Multimedia component 13 [file mmc13.docx]
