# Supplementary material for: The impact of non-environmental factors on the chemical variation of Radix Scrophulariae
Source: Heliyon. 2024 Jan 12;10(2):e24468. doi: 10.1016/j.heliyon.2024.e24468 (PMC10831622; doi:10.1016/j.heliyon.2024.e24468)
Supplement: Multimedia component 14 [file mmc14.docx]

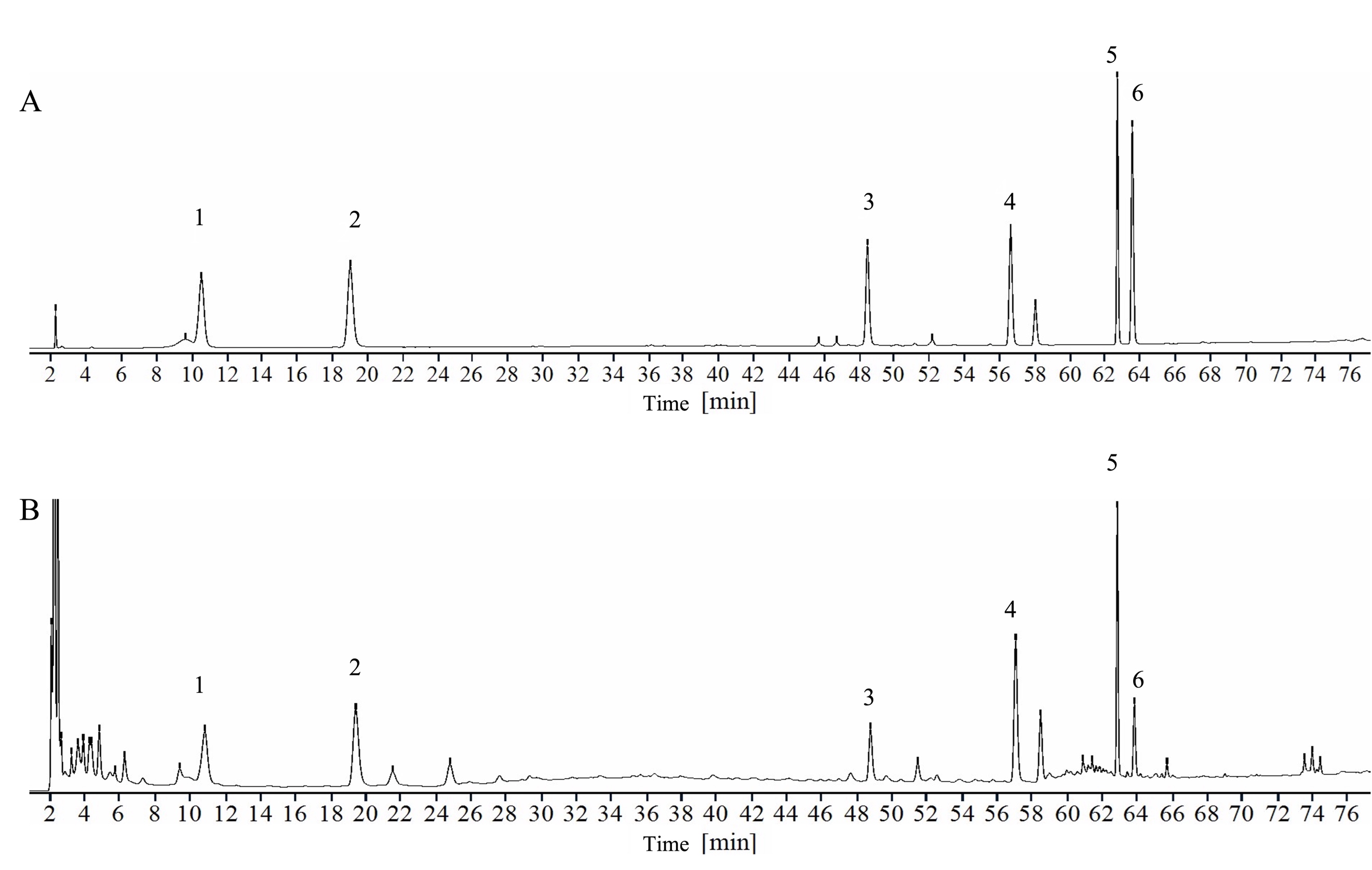


Fig. S2. The chromatogram of all standards applied in this study. A: chromatogram of all standards; B: chromatogram of sample. 1: aucubin, 10.460 min; 2: harpagide, 18.985 min; 3: acteoside, 48.391 min; 4: angoroside c, 56.532 min; 5: harpagoside, 62.661 min; 6: cinnamic acid, 63.568 min.
